# A review of nanomaterials in osteoarthritis treatment and immune modulation

**DOI:** 10.1093/rb/rbaf048

**Published:** 2025-06-04

**Authors:** Wei Deng, Tianshu Wang, Lei Li, Xuanyu Xiao, Yuanyuan Xu, Qiujiang Li, Qingsong Zhou, Yong Yin, Hongsheng Yang, Kai Gong, Yue Zhou, Yunbing Wang

**Affiliations:** Department of Orthopedics, The Third Affiliated Hospital of Chengdu Medical College, Chengdu Pidu District People's Hospital, Chengdu, Sichuan 611730, China; National Engineering Research Center for Biomaterials & College of Biomedical Engineering, Sichuan University, Chengdu, Sichuan 610065, China; National Engineering Research Center for Biomaterials & College of Biomedical Engineering, Sichuan University, Chengdu, Sichuan 610065, China; National Engineering Research Center for Biomaterials & College of Biomedical Engineering, Sichuan University, Chengdu, Sichuan 610065, China; National Engineering Research Center for Biomaterials & College of Biomedical Engineering, Sichuan University, Chengdu, Sichuan 610065, China; Department of Orthopedics, Sichuan Provincial People's Hospital, School of Medicine, University of Electronic Science and Technology of China, Chengdu 610072, China; Department of Orthopedics, The Third Affiliated Hospital of Chengdu Medical College, Chengdu Pidu District People's Hospital, Chengdu, Sichuan 611730, China; Department of Orthopedics, The Third Affiliated Hospital of Chengdu Medical College, Chengdu Pidu District People's Hospital, Chengdu, Sichuan 611730, China; Department of Orthopedics, The First Affiliated Hospital of Chengdu Medical College, Chengdu, Sichuan 610500, China; Department of Orthopedics, The First Affiliated Hospital of Chengdu Medical College, Chengdu, Sichuan 610500, China; Department of Emergency Medicine, West China Hospital, Sichuan University, Chengdu, Sichuan 610041, China; West China School of Nursing, Sichuan University, Chengdu, Sichuan 610041, China; Disaster Medical Center, Sichuan University, Chengdu, Sichuan 610041, China; Nursing Key Laboratory of Sichuan Province, Chengdu, Sichuan 610041, China; National Engineering Research Center for Biomaterials & College of Biomedical Engineering, Sichuan University, Chengdu, Sichuan 610065, China

**Keywords:** osteoarthritis, nanomaterials, immune response, treatment mechanisms

## Abstract

Osteoarthritis (OA) is a highly prevalent degenerative cartilage disease globally. The medical community has recognized it as one of the major public health problems today. Nanomaterials are considered the most promising avenue for OA treatment because they exhibit unique physicochemical properties such as high catalytic activity, bio-enzyme-like reaction kinetics, and modulation of joint immune responses. Besides, nanomaterials can exert higher targeting to improve therapeutic efficacy and reduce side effects. These unique advantages have led to the widespread development of nanomaterials for OA treatment, and they are gradually seeing their most prosperous moment. A timely and comprehensive review of OA pathogenesis-immunomodulation-therapeutic efficacy from a nanomaterials perspective would greatly broaden this research area. This review summarizes the recent advances in nanomaterials for OA treatment. Finally, the main challenges and opportunities for nanomaterials to modulate the immune system for OA treatment are discussed.

## Graphical abstract

**Figure:**
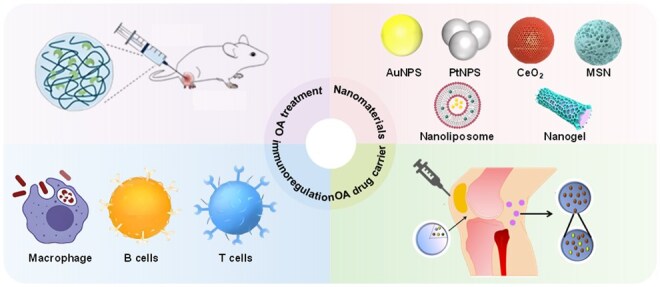

## Introduction

Osteoarthritis (OA) is the most prevalent degenerative joint disease, affecting both small joints in the hands [1–3] and large joints such as the knees [4–6] and hips [7–9]. Numerous factors contribute to the onset of OA, including aging [10], obesity [11], increased biomechanical load [12] and external injuries [13] (such as fractures, single or recurrent sprains, and anterior cruciate ligament tears [14]). OA currently affects approximately 250 million individuals worldwide, with severe cases potentially leading to disability [15, 16]. Research indicates that the pathological process of OA is primarily governed by complex immune responses [17], involving the interaction of immune cells [18], inflammatory mediators [19] and cytokines [20], which ultimately result in pathological changes to the joint synovium [21] and cartilage [22], establishing a vicious cycle. Therefore, modulating these immune responses and processes is the key to treating OA.

The traditional treatments for OA are mainly systemic administration of drugs [23] or end-stage total joint replacement-type surgery [24]. However, systemic administration of drugs has strong toxic side effects on other organs (non-steroidal anti-inflammatory drugs may cause cardiovascular and gastrointestinal burdens, and opioid-strength painkillers carry the risk of addiction). Total joint replacement can cause large trauma to the organism. Currently, the main method of treating OA by modulating the function of the nodal immune system is to inject drugs (dexamethasone, interleukin-1 receptor antagonist (IL-1Ra), anti-inflammatory cytokine IL-4, etc) into the diseased joints, which is a more suitable method than systemic administration of drugs that have a high risk of systemic toxicity and poor bioavailability to the joints [25]. However, this method of drug delivery still suffers from insufficient drug residence time [26], high toxicity [27], high drug concentration and high cost, but poor efficacy. In addition, long-term circulating injection of drugs will affect patients' emotions. Therefore, efforts are being made to develop drugs that precisely target the OA immune system and have a long half-life. Nanoparticles (NPs) exhibit good potential for applications in drug delivery [28], tissue repair [29] and inflammation modulation [30] due to their unique physicochemical properties. For example, NPs, when used as drug carriers, enable drugs to act with greater targeting and biocompatibility, improving therapeutic efficacy and reducing side effects [31]. Meanwhile, some NPs can be used as therapeutic agents to promote OA tissue regeneration [32] or inhibit inflammatory responses directly [33]. By modulating the properties of nanomaterials, smart materials can be better designed to meet the therapeutic needs of OA. In conclusion, NPs-based means of treating OA are considered one of the most promising avenues for the future [34].

Although nanomaterials have been extensively researched and applied in OA treatment, the role played by the immune system in the course of OA cannot be ignored. During OA treatment, nanomaterials and drugs can regulate the local microenvironment and influence the activity of immune cells and cytokine secretion, which breaks the vicious cycle of inflammatory response. Recent reviews have focused on summarizing risk factors [23], mechanisms [35], therapeutic agents and their side effects [36], typical symptoms and diagnostic methods for developing OA [37]. There is a lack of cohesive reports on the progress of research on treating OA by modulating the immune system through nanomaterials. In context, a systematic summary of the immune mechanisms of OA pathology and the therapeutic applications of nanomaterials in OA can help researchers gain a clearer understanding of the principles of the interaction between nanomaterials and the immune system, and assist in designing novel nanomedicines for OA treatment.

**Figure 1.**
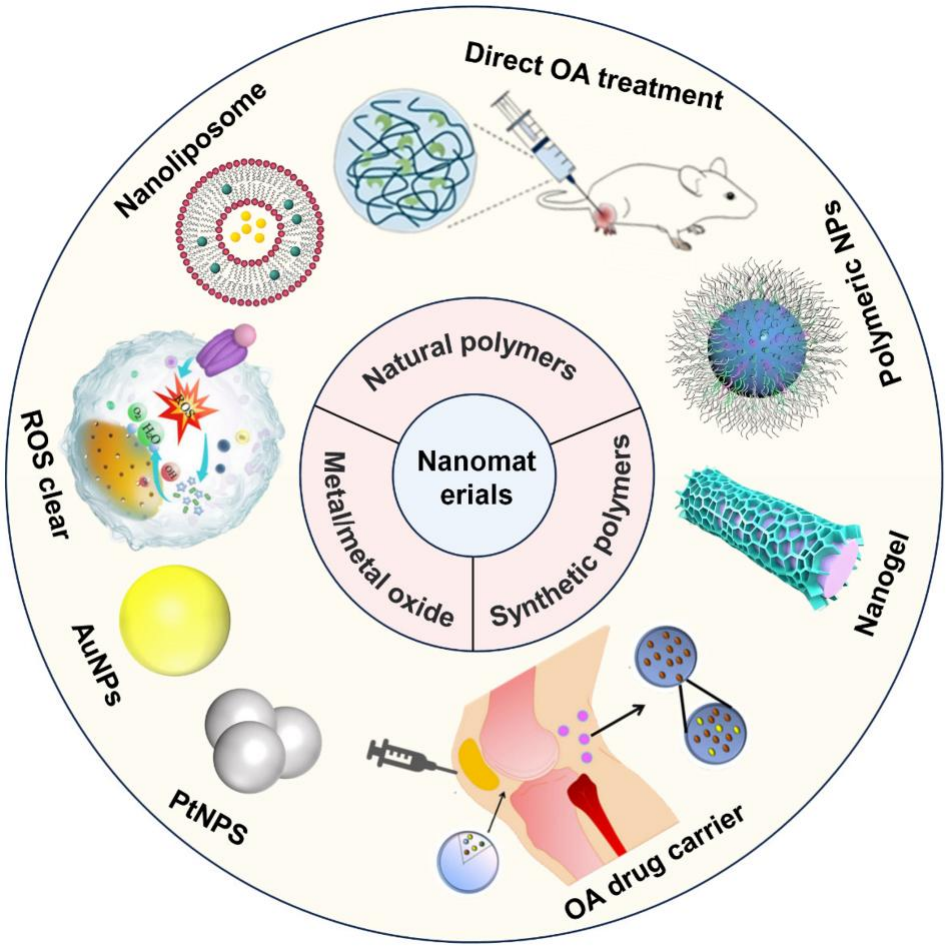
Nanomaterials for OA treatment and their modulation of the immune system.

In this review, we mainly focus on the progress of nanomaterials in OA treatment, providing insights into their various properties that modulate the immune system (Figure 1). First, we introduced the immunopathogenesis of OA, emphasizing the application of nanomaterials in OA treatment and immune response. Then, the advantages of different nanomaterials (metals, metal oxides, synthetic polymers and natural polymers) and typical examples are described in detail. Besides, the specific mechanism of the immune system in OA treatment was briefly discussed. Finally, we explore the challenges, opportunities and advantages of nanomaterials to modulate the immune system for OA. We believe that exploring the immune mechanism and the innovative application of nanomaterials can bring new hope for OA treatment. We hope this review will provide new ideas for researchers to study the role played by the immune system in OA and the development of nanomedicines.

## Pathology and immunologic mechanisms of OA

OA is a prevalent chronic disease caused mainly by cartilage wear and tear due to aging or obesity. Clinically, OA is commonly found in the knees, hands and hips [38]. The clinical symptoms of OA are characterized by chronic pain, joint stiffness and narrowing of the joint space, which may be disabling in severe cases [39]. This section introduces the specific pathogenesis of OA and focuses on the mechanism of the immune system.

### Pathological mechanisms of OA

One of the most important factors in the development of OA is damage to bone or cartilage, leading to the destruction of joint structure and imbalance of force balance. High joint loading due to obesity increases the risk of developing structural OA [40]. Recent research suggests that visceral obesity may be associated with pain symptoms in OA [41]. The prevalence of OA increases with age, and telomere attrition, altered intercellular communication, and cellular senescence promote the development of OA [42].

**Figure 2.**
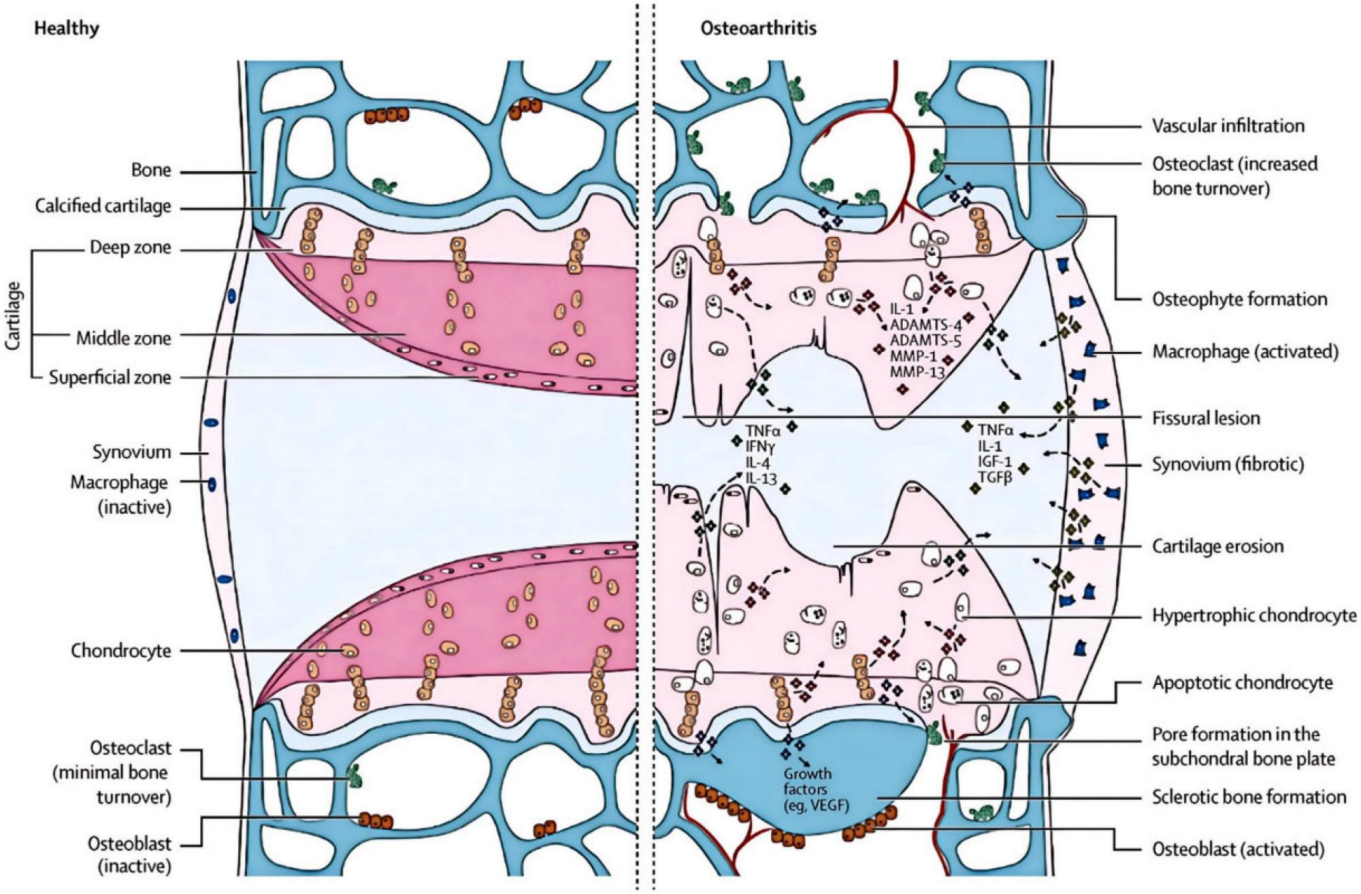
Signaling pathways and structural changes in OA development. Reprinted with permission from Ref. [16].

OA involves the entire joint, including ligaments, synovium, joint capsule, articular cartilage, subchondral bone, meniscus and surrounding muscles [43]. The pathogenesis of OA includes several pathological changes in cartilage, synovium and subchondral bone. These changes destroy joint structures and exacerbate inflammation and metabolic dysregulation in a vicious cycle. OA begins with a change in the properties of the cartilage. Damage to articular cartilage begins with surface erosion, progressively deepens through cartilage and bone fractures, and eventually leads to expansion of the calcified cartilage zone. During cartilage repair, hypertrophic chondrocytes have increased synthetic activity, proinflammatory mediators and matrix degradation products, leading to chondrocyte dysfunction and stimulation of synovial proliferation and proinflammatory responses. Synoviocytes release pro-inflammatory products accompanied by tissue hypertrophy and increased vascularity. At the same time, there is an increase in bone conversion, vascular infiltration, remodeling and repair processes associated with subchondral bone marrow lesions in the subchondral bone. Under the influence of inflammatory substances, the joint margins develop abnormally, forming osteophytes (Figure 2) [16]. OA is a heterogeneous disease whose pathogenesis is associated with cellular senescence, metabolic alterations [44], mechanical damage [45] and immune system involvement.

### Immune system involvement in OA

As a chronic inflammatory disease, the immune system plays an important role in the pathogenesis of OA. Immune system-mediated chronic low-grade inflammation leads to synovial changes, cartilage degeneration and bone remodeling. The immune system that mediates the development of inflammation includes the innate and adaptive immune systems. In OA, the innate immune system is the main research object. During the progression of OA, the number of immune cells, such as macrophages and T cells, increases significantly and secretes inflammatory factors with other cells to promote inflammation [46]. Synovitis is a typical feature of OA because macrophages are located mainly in the synovium. In addition, pro-inflammatory mediators are also produced at sites such as articular cartilage and subchondral bone. In OA, inflammatory mediators such as cytokines, chemokines and growth factors interact to cause pain and promote inflammation [47]. This section details the immune cells and inflammatory mediators that primarily mediate OA.

#### Key immune cell types

The key immune cells involved in the progression of OA include macrophages and T-lymphocytes, and the level of their infiltration is strongly correlated with the course of the disease and painful symptoms.

##### Macrophages

**Figure 3.**
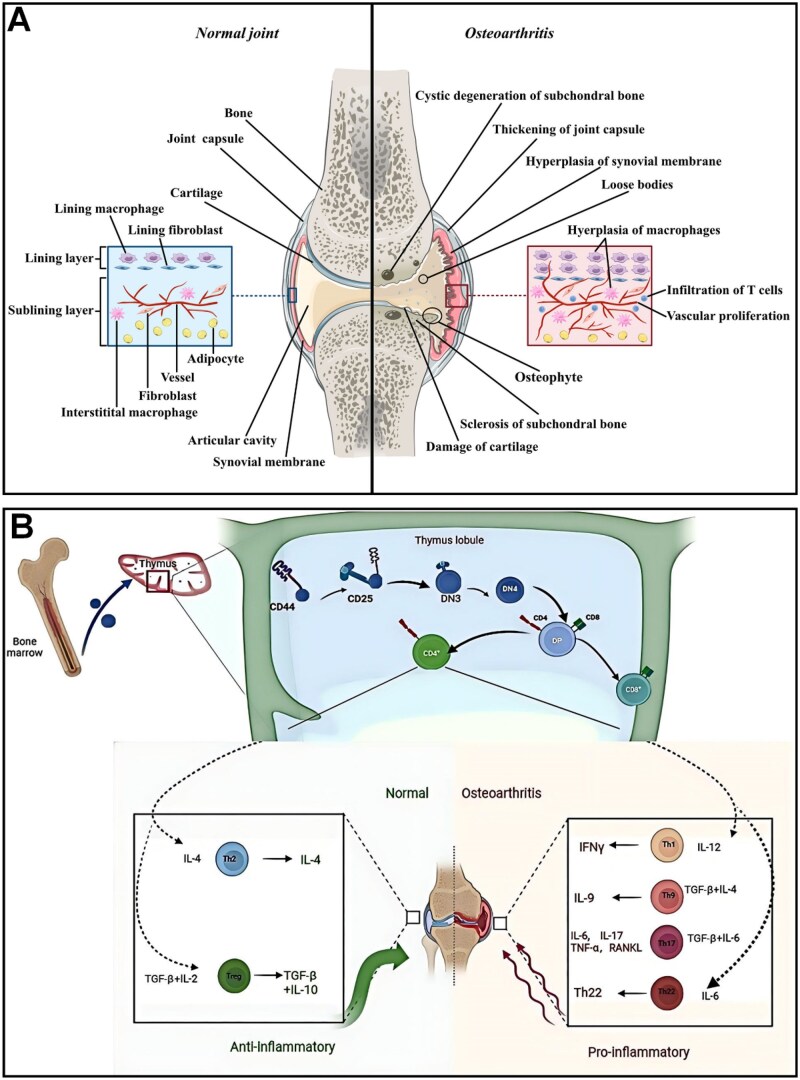
(**A**) Comparison of normal and OA joints. Reprinted with permission from Ref. [48]. (**B**) Schematic representation of the effects of different subsets of T cells and cytokines on the course of OA. Reprinted with permission from Ref. [49].

Macrophages are found primarily in the synovial membrane and consist of two layers of cells (Figure 3A) [48]. Synovial fibroblasts and macrophages constitute the inner synovial layer and maintain joint homeostasis. The outer layer composed of different types of connective tissue coordinates joint function. In the presence of OA, the number of synovial macrophages in the inner layer was significantly increased and correlated with the severity of OA [50]. Macrophages are categorized into three phenotypes based on their function: unstimulated macrophages (M0), pro-inflammatory macrophages (M1) and anti-inflammatory macrophages (M2). M0-type macrophages can be activated and converted to M1- or M2-type macrophages after receiving certain stimuli in the microenvironment. M1-type macrophages are stimulated to produce pro-inflammatory factors such as interleukin 1β (IL-1β), IL-6 and IL-12. In contrast, M2-type macrophages are stimulated to produce anti-inflammatory factors such as transforming growth factor beta (TGF-β) and IL10. However, the M1 and M2 types of macrophages are the only two macrophage types that differentiated *in vitro* in past studies and are not convincing in a true OA disease system. Macrophages in normal and disease (OA and rheumatoid OA) differ markedly and exist in multiple subpopulations. Several reports have investigated the effect of these two types of macrophages on OA by varying the M1/M2 ratio [48]. For example, by reverse transcription PCR, Liu *et al.* quantified synovial fluid and peripheral blood CD11c (M1-type macrophage marker) and CD206 (M2-type macrophage marker) data from 80 patients with normal knee OA. The results showed that the ratio of M1 to M2 macrophages was significantly higher in patients with knee OA than in controls, and was significantly positively correlated with the level of Kellgren–Lawrence classification in knee OA [51]. Some researchers recognized the proliferation of M2-type macrophages as a potential target for OA treatment. Based on this, Ma *et al.* used chondroitin sulfate ChS to mimic the anti-inflammatory component of M2-type macrophages, effectively stopping the vicious cycle in the pathological environment of OA. Anti-inflammatory components secreted in the OA environment, successfully mimic some of the anti-inflammatory functions of M2 macrophages and effectively stop the vicious cycle in the pathological environment of OA [52]. Besides, Wood *et al.* compared synovial macrophages from OA and inflammatory arthritis (IA) by RNA sequencing and found that one OA subset was similar to IA and the other was different. They termed OA different from IA as classic OA (cOA) and similar to IA as inflammatory OA (iOA). Flow cytometry demonstrated that the number of synovial macrophages was higher in the iOA than in the cOA group, which may represent a different disease mechanism [53]. Macrophages exacerbate OA mainly by producing pro-inflammatory factors and influencing intercellular interactions. Huo *et al.* found that macrophage-secreted CX3XL1 was positively correlated with pain and physical disability in patients with OA [54]. Daghestani *et al.* found that macrophage markers in the synovial fluid of patients with OA (sCD163 and sCD14) were positively correlated with the joint space in OA stenosis and the severity of bony encumbrances was positively correlated [55]. In addition to secreting inflammatory mediators, synovial macrophages interact with cells. Danalache *et al.* studying cartilage samples from OA patients found that matrix metalloproteinases (MMP) disrupt pericellular matrix (PCM) protection and signaling within chondrocytes and exacerbate OA [56].

##### T cells

T cells are the major cellular component of the adaptive immune system, originating from hematopoietic stem cells and differentiating, developing and maturing in the thymus. The disease microenvironment can activate T cells and produce cytokines and inflammatory mediators. Numerous studies have shown that activated T cells and their immune response are closely related to bone loss and destruction in OA. T cells influence the course of OA by differentiating into different subpopulations and secreting different cytokines (Figure 3B) [49]. Sae *et al.* found that aberrant expression of pro-inflammatory factors such as TNF-α by T cells enhances osteoclast activity and contributes to bone loss and destruction [57]. Noriko *et al.* demonstrated that different types of T cells have different effects on osteoclasts. CD4+T, Th1 and Th17 cells enhance osteoclast activity, while CD8+T cells and Treg inhibit osteoclast activity [58]. CD4+T cells influence the OA process by secreting TNF-α and IL-6. De *et al.* identified a CD4+T cell population that may contribute to joint inflammation [59]. Besides, T cells also affect enzymatic reactions. Platzer et al. using enzyme-linked immunosorbent assay found that activated T cells induced the release of MMP, disrupted PCM and destroyed cartilage [60].

##### Interactions between immune cells and effects on OA

In the pathological process of OA, immune cells interact in complex ways and contribute to OA's inflammatory response and cartilage damage. For instance, dendritic cells (DCs), a heterogeneous group of antigen-presenting cells involved in innate and adaptive immune responses [61], interact with T cells and other immune cells in the OA pathological environment [62]. In OA patients, the DC subgroups in synovial fluid mainly consist of dendritic cell (cDC) and plasmacytoid dendritic cell (pDC). The synovial fluid of OA patients is rich in mature cDCs and pDCs through the release of high-level inflammatory mediators, promoting Th1 and Th17 cell differentiation, directly or indirectly via inflammatory mesenchymal stem cells (MSCs), inhibiting chondrogenesis, and inducing cartilage degeneration [63]. DCs also participate in the initiation and maintenance of OA by expressing high-level Toll-like receptors (TLRs) and secreting large amounts of pro-inflammatory cytokines. Besides, some immature DCs can modulate the immune response in inflamed joints by releasing IL-10 [64], promote the proliferation of regulatory T cells (Treg) and stimulate the chondrogenic differentiation of MSCs, thereby inhibiting the inflammatory response [65]. In addition, B cell differentiation and function rely on T cell help, and in turn, differentiated B cells can influence T cells. This interplay is significant in OA's pathological process. B cells can differentiate into plasma cells (PCs) via both T cell-dependent (T-dep) and TLR-dependent (TLR-dep) pathways, subsequently secreting immunoglobulins (IgM, IgA, IgG). Additionally, B cells regulate T cell functionality through antigen presentation and cytokine secretion [66]. For example, B cells express CD86, which binds to the CD28 receptor on T cells, in addition to the antigen-specific signal, providing the necessary second signal for the complete activation of T cells [67]. This interaction promotes T cell proliferation and cytokine production, influencing B cell activation and thereby facilitating inflammation and the intensification of cartilage degeneration caused by synovitis [68, 69].

#### Role of cytokines, chemokines and complement

Synovitis is a typical symptom of OA. IL-1β and TNF-α are two major pro-inflammatory cytokines contributing to cartilage homeostasis' progression toward cartilage degradation [70].

**Figure 4.**
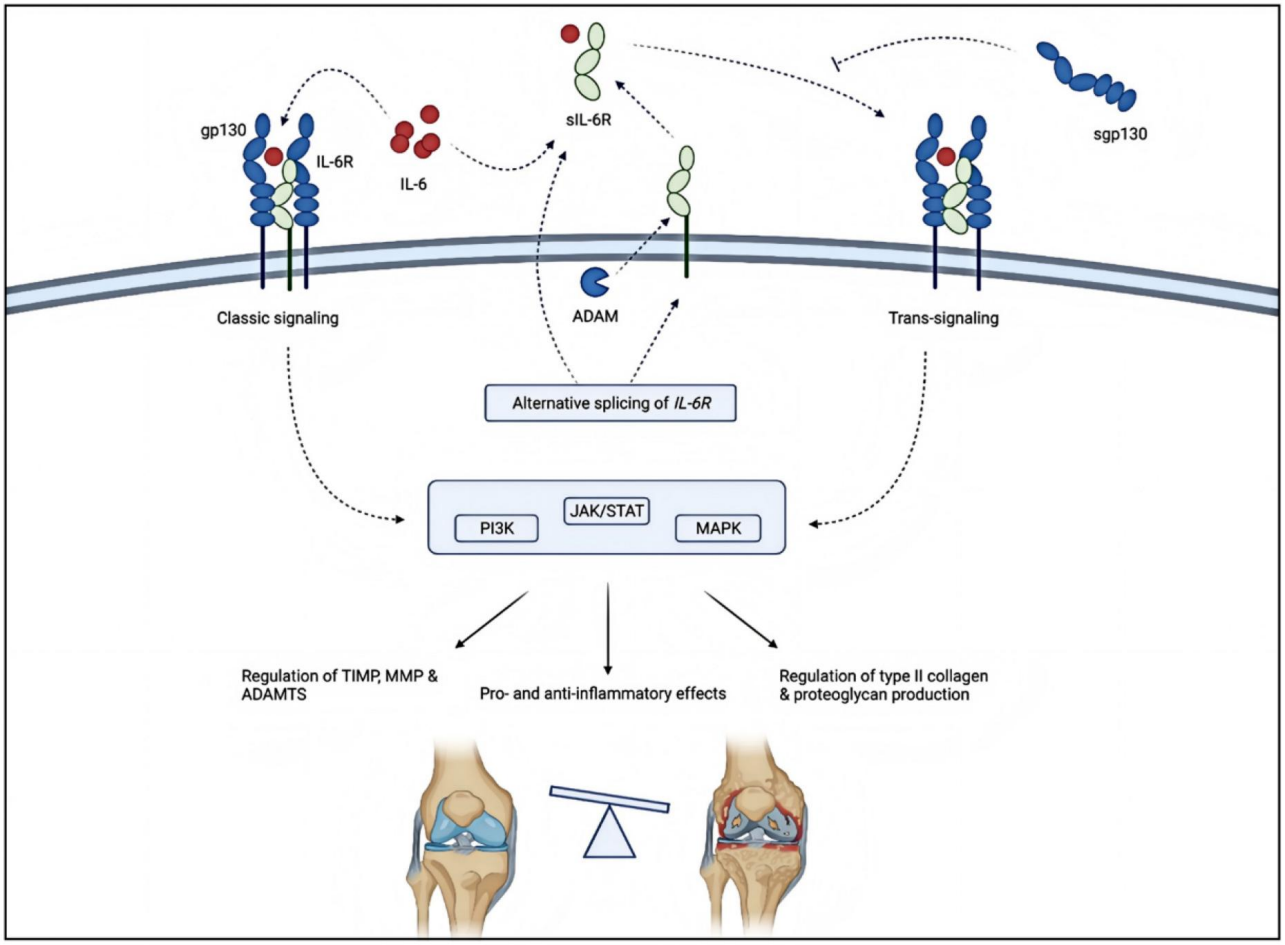
Schematic diagram of the mechanism of action of IL-1β in OA. Reprinted with permission from Ref. [71].

IL-1β is a member of the IL superfamily and functions by binding to the interleukin 1 receptor (IL-1RI). In OA patients, IL-1β is overexpressed in synovial tissue and articular cartilage, while IL-1Ra is downregulated. The nuclear factor κ light chain enhancer (NF-κB) of activated B cells is one of the most important signaling pathways for IL-1β-mediated OA. The NF-κB signaling pathway promotes IL-6, TNF-α secretion, and increased synthesis of MMP and disintegrin-like metalloproteinases with platelet-responsive protein motifs (ADAMTS), which disrupt collagen and leads to proteoglycan degradation. Besides, the synthesis of various chemokines increases and promotes the involvement of inflammatory cells in the inflammatory response. Inflammatory cells continue to secrete cytokines such as IL-1β, establishing a vicious cycle of OA. In addition, the mitogen-activated protein kinase (MAPK) signaling pathway is another important signaling pathway in OA. MAPK mainly comprises p38MAPK, c-Jun N-terminal kinase (JNK) and extracellular signal-regulated kinase (ERK). By activating these kinases, IL-1β inhibits collagen synthesis, reduces extracellular matrix secretion and downregulates the expression of genes for matrix component synthesis, leading to apoptosis of chondrocytes. At the same time, this signaling pathway also stimulates the secretion of pro-inflammatory factors to enhance IL-1β activity, creating positive feedback [71]. We summarized the mechanism of action of IL-1β (Figure 4) [71]. Researchers considered treating OA by inhibiting the effect of IL-1β but did not achieve the expected effect. It is possible that IL-1β is only part of the vicious cycle of OA disease and that other cytokines may have compensatory mechanisms for IL-1β.

Tumor necrosis factor-α (TNF-α) is a pro-inflammatory cytokine and a potent anti-tumor effector. TNF-α can be produced by lymphocytes, osteoblasts and adipocytes, promoting T-cell differentiation and proliferation and stimulating chemotaxis and phagocytosis of immune cells. During OA, TNF-α is produced mainly by synoviocytes and chondrocytes and is involved in structural damage and the regulation of pain [72]. TNF has two specific receptors on the cell membrane, TNF receptor I (TNFRI) and TNFRII. The expression of TNFRI is higher in synoviocytes and chondrocytes of OA. TFN-α and IL-β mediate the same signaling pathways and act synergistically. Thus, TNF-α similarly stimulates the production of IL-6, IL-8 and other pro-inflammatory cells, increases NO production and blocks proteoglycan and collagen synthesis. Meanwhile, TNF-α can act on collagenase and MMP to mediate ECM degradation. In addition, Patel *et al.* showed elevated mRNA expression of TNF-α converting enzyme (TACE) in the cartilage of OA patients. TACE can mediate the maturation of TNF-α precursors and may be one of the targets for OA treatment.

Other pro-inflammatory cytokines (including IL-6, IL-15, IL-17, leukemia inhibitory factor (LIF), IL-18 and IL-21), as well as anti-inflammatory cytokines (including IL-4, IL-10 and IL-13), also play a role in chondrocyte metabolism and the development of OA. IL-6 is a pro-inflammatory factor that increases inflammatory responses and promotes OA repair through anti-inflammatory effects. The main sources of IL-6 in OA are chondrocytes, osteoblasts and synoviocytes [73]. IL-6 activates classical and trans-signaling by binding to the membrane-bound IL-6 receptor (mbIL-6R) or soluble IL-6 receptor (sIL-6R), respectively [74]. Trans signaling activates pro-inflammatory pathways primarily through glycoprotein 130, whereas classical signaling may promote anti-inflammatory and regenerative processes. Li *et al.* showed that IL-6 concentrations in synovial fluid were elevated in patients with OA and correlated with pain and disease severity [75]. IL-6 is influenced by the concentrations of sIL-6R and soluble glycoprotein 130, which determine the balance between trans and classical signaling. The pro-inflammatory cytokine IL-15 is a potential biomarker in the early diagnosis of OA. It has high concentrations in synovial fluid in the early stages of OA, correlates with patient-reported pain severity, enhances MMP production and affects cartilage matrix degradation [76]. IL-17A is the most studied member of the IL-17 family and is produced primarily by Th17 [77]. In OA, Na *et al.* showed that IL-17A resulted in a significant increase in the expression of MMP1, MMP3, MMP13 and monocyte chemotactic protein-1 (MCP-1) and a significant decrease in TIMP3 and COL2A1. Besides, the expression of SOX9, a master anabolic chondrogenic transcription factor, was reduced in an IL-17-dependent manner. TIMP3 is an anabolic marker, whereas COL2A1 and SOX9 are markers of cartilage formation, suggesting that IL-17 is a key promoter of cartilage destruction [78]. IL-4 is also present in the pathological environment of OA and is mainly secreted by Th2 cells, eosinophils, basophils and mast cells. It has been shown that IL-4 concentrations in serum and synovial fluid are significantly higher in patients with OA, possibly related to the increased number of CD4+T cells infiltrating the synovium [79]. IL-10, mainly produced by immune and chondrocyte cells, activates the JAK-STAT kinase pathway and exerts chondroprotective, anti-apoptotic and anti-inflammatory effects. IL-4 and IL-10 can stimulate the synthesis of type II collagen and aggregated proteoglycans while inhibiting MMP synthesis and reducing the expression of pro-inflammatory factors such as TNF-α, IL-6 and IL-12.

Chemokines are usually a class of small peptides of 8–12 kDa, consisting of C, CC, CX and CXC four families. Chemokines act through G protein-coupled receptors on the cell surface and their functions and mechanisms may differ in different joints [80]. They drive cell aggregation in OA. For example, it induces macrophage infiltration and osteoclast activation at the subchondral bone, resulting in osteoporosis. Cartilage tissue hinders proteoglycan synthesis, promotes ECM breakdown leading to apoptosis and inhibits cell proliferation. In synovial tissue, it prompts the transformation of monocytes into macrophages, exacerbating the local inflammatory response [81]. CC family chemokines are widely present in OA. For example, CCL2 (MCP-1) is produced by various cells and binds to CCR2 to mediate the migration of monocytes and T lymphocytes. One group found that CCL2 levels were significantly elevated in the synovial fluid of patients with knee and ankle injuries, and their levels correlated with knee imaging changes and clinical symptoms [82]. This result demonstrates that CCL2 has a pro-inflammatory and cellular infiltration role in the development of OA and may also be associated with pain and neuroinflammation. The CCL2 may also induce MMP-3 production by chondrocytes *in vitro* and promote matrix degradation [83]. In addition, other CC family members, such as CCL3, CCL4 and CCL5, are also expressed and upregulated in OA and are involved in cartilage metabolism and inflammatory processes. Also, the expression of CCL19 and CCL21 correlates with the severity of OA and can be used as potential biomarkers. CXCL12 is one of the most widely studied chemokines in tissue repair and binds to the receptor CXCR4 to mobilize MSCs to the injury site to aid bone damage repair [84]. CXCL12 was shown to promote endogenous chondrogenic progenitor cell recruitment and tissue repair and ameliorate cartilage defects in an experimental model in OA patients *in vivo*. In addition, CXCL12 induced adult articular chondrocytes to produce degradative enzymes such as MMP13 to promote matrix catabolism and IL-6 production by synovial fibroblasts [85]. The researchers established a guinea pig model of spontaneous OA and blocking CXCR4 signaling with a drug reduced cartilage degeneration and the expression of associated inflammatory and degradative mediators [86]. Recent studies have revealed that CXCR2 signaling has a homeostatic function in maintaining the normal phenotype of chondrocytes and protecting degenerating chondrocytes [87].

**Figure 5.**
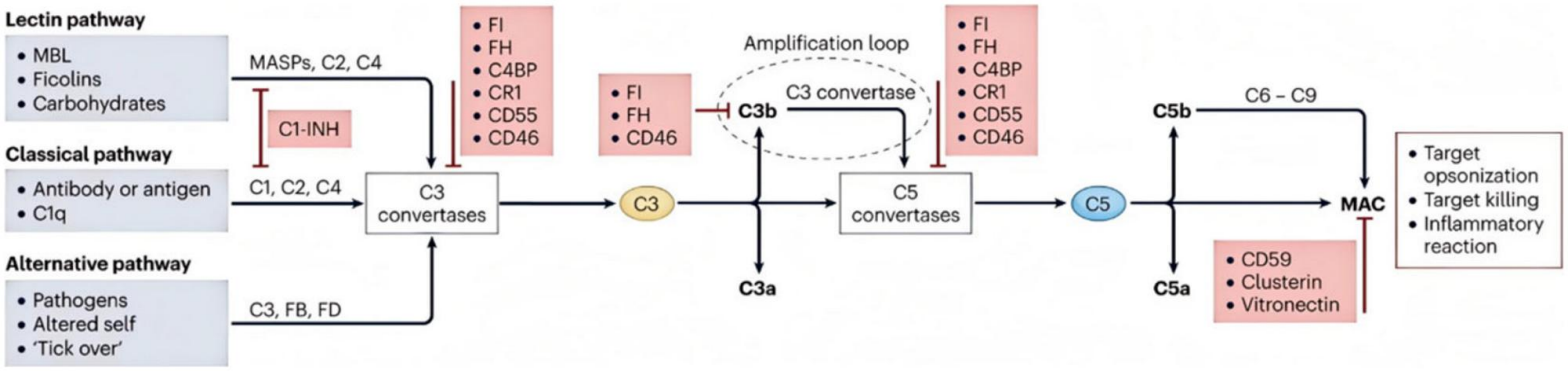
Schematic representation of the complement cascade reaction to generate the pro-inflammatory mediators C3a and C5a allergenic toxin MAC (F: factor; MASP1/2: MBL serine proteases 1 and 2; MBL: mannose-binding lectin). Reprinted with permission from Ref. [88].

The complement system plays a crucial role in the immune response and is activated through three separate initiation pathways (classical, alternative and lectin pathways). The activated C3 and C5 converting enzymes initiate the complement cascade, producing C3a and C5a allergenic toxins and the membrane attack complex (MAC) (Figure 5) [88]. The C3a and C5a allergenic toxins attract pro-inflammatory leukocytes and promote an inflammatory response. They also attract immune cells to the site of infection or injury to remove pathogens and damaged tissue. MAC is the ultimate effector of the complement cascade reaction, directly attacking bacterially and virally infected cells and causing cell death. In some cases, MAC can induce pro-inflammatory cell conductance, leading to further inflammatory responses and tissue damage by activating signaling pathways [89]. The complement system is involved in immune defense, inflammatory response and tissue damage through various mechanisms. Wang *et al.* found by experimentation and observation of genetically defective mouse and human samples that cartilage-released components in OA may trigger a complement cascade response and lead to dysregulated gene expression. Uncontrolled complement activation leads to MAC formation in chondrocytes, resulting in cell death or the production of inflammatory mediators that promote the course of OA [90]. The complement system is activated in early OA and persists into the late stages. Low-grade inflammation promotes the production of allergenic toxins C3a and C5a [91]. The activation of the complement system is an important part of the vicious cycle of OA, and it continues to destroy tissue.

#### Modulatory effects of gut microbiota on OA immune responses

In recent years, the gut microbiota's role in modulating OA immune responses has gained attention, with new research indicating it may influence OA's pathological process via the ‘gut-joint axis’ [69]. Gut microbiota may affect OA immune responses by altering gut barrier function through its metabolic products. For example, intestinal dysbiosis can increase intestinal barrier permeability, allowing bacterial products and pro-inflammatory factors to enter the systemic circulation and promote inflammation at bone joints [92]. Clinical studies have shown that the concentration of lipopolysaccharide (LPS) in the serum and synovial fluid of OA patients is positively correlated with the severity of knee joint damage and synovial inflammation in OA patients [93]. Gut microbiota dysbiosis may increase gut permeability, enabling bacterial products like LPS to enter the bloodstream [94]. LPS exacerbates joint inflammation by activating the TLR4 pathway [95], prompting macrophages and synovial cells to release pro-inflammatory factors [96]. In contrast to LPS, another class of intestinal flora metabolites, short-chain fatty acids (SCFAs), have significant immunomodulatory properties that protect the intestinal barrier from intestinal flora dysbiosis. SCFAs can reduce gut permeability by specifically modulating the expression of mucin genes in gut epithelial cells and activating AMP-activated protein kinase (AMPK) to promote the assembly of tight junctions [97].

Gut microbiota dysbiosis can also lead to the activation of the innate immune system. For instance, LPS can activate M1-type macrophages, induce the secretion of pro-inflammatory cytokines and cause secondary inflammation in articular cartilage [98]. Besides, an increase in intestinal streptococci may also affect macrophages and exacerbate OA inflammation [99]. This may occur as *Streptococcus*-derived metabolites and membrane vesicles can traverse the gut barrier to activate synovial macrophages, triggering systemic low-grade inflammation and worsening joint inflammation. Neutrophils, as the body's first line of microbial defense and synergistic partners with macrophages, may also be influenced by gut microbiota, thereby affecting OA progression [100]. Some studies have found that bacterial peptidoglycan (PGN), a gut microbiota metabolite, can enhance neutrophil function through the nucleotide-binding and oligomerization domain 1 (NOD1) receptor [101], while SCFAs can inhibit inflammation by reversing neutrophil recruitment [102]. The interactions between gut microbiota products and neutrophils are likely to impact OA progression. However, no studies have confirmed the potential link between gut microbiota, neutrophils and OA.

## Application of nanomaterials in OA treatment

Currently, traditional drug therapy for OA has shortcomings such as high toxicity, systemic toxicity and poor therapeutic effect. Many researchers have used nanotechnology to improve OA treatment efficacy. The unique dimensionality of nanomaterials gives them the potential for precisely targeted therapy (utilizing photosensitive, thermal and magnetic properties) and reduced drug toxicity. This section describes specific applications of nanomaterials in OA treatment.

### Types of nanomaterials for OA treatment

Different types of NPs have various properties that affect their drug release, delivery rate and efficacy as drug carriers for OA treatment. Therefore, selecting specific kinds of NPs is crucial for OA treatment. This section introduces different kinds of NPs and their classical use cases in OA treatment. We also summarize a selection of representative application cases (Table 1).

**Table 1.** Classic cases of different types of NPs used for OA treatment in recent years

Type	NPs name	Morphology	Trigger mechanism	Loaded drug/acting ingredient	Treatment time	Model	Advantages	Refs.
Metal/metal oxide nanomaterials	Gd_2_(CO_3_)_3_@PDA(Hes)-PEG-DW	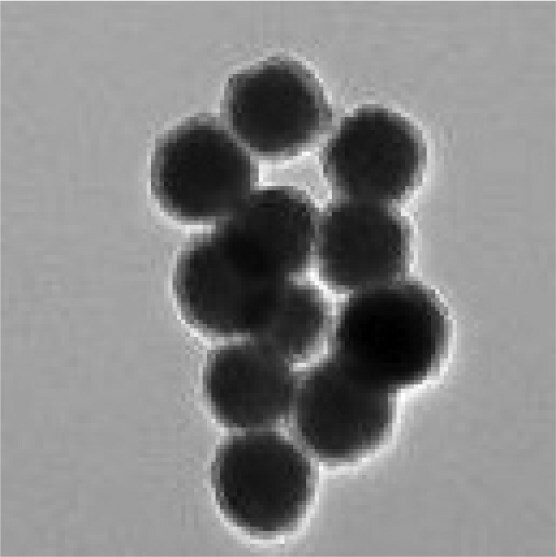	Slowing down inflammation through TLR-2/NF-κB/Akt signaling	Hes	3 d	ACLT mice	Low side effects, cartilage-specific	[103]
	Bai@FA-UIO-66-NH_2_	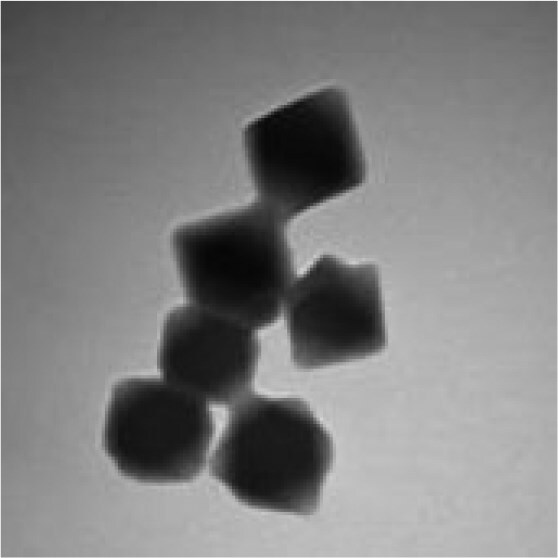	Macrophage polarization, ROS clearance	Bai	72 h	ACLT rats	Improved bioavailability	[104]
	Ta-NH_2_ NPs	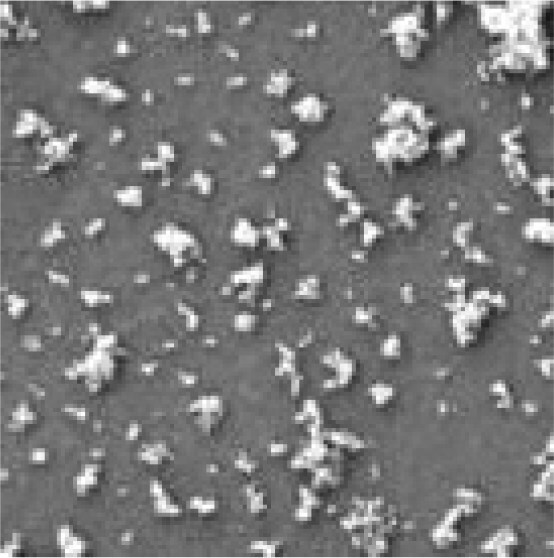	ROS clearance	Ta	8 weeks	MIA rats	Long-term stable therapeutic effects	[105]
	NIR-responsive molybdenum (Mo)-based nanoclusters	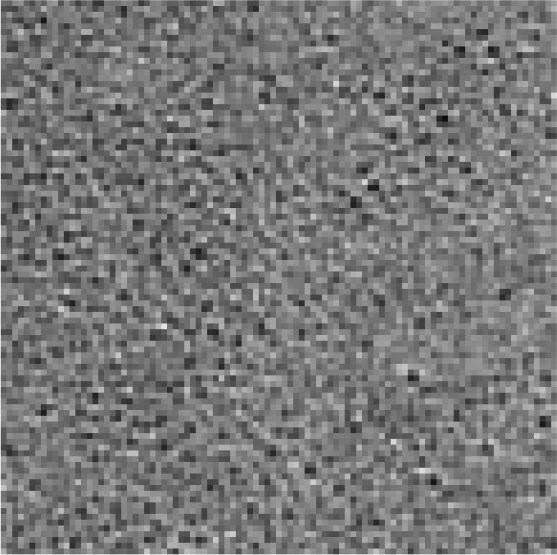	ROS clearance	Mo	3 d	MIA mice	Photothermal properties enhance the removal	[106]
Metal/metal oxide nanomaterials	TP-Au@PCN	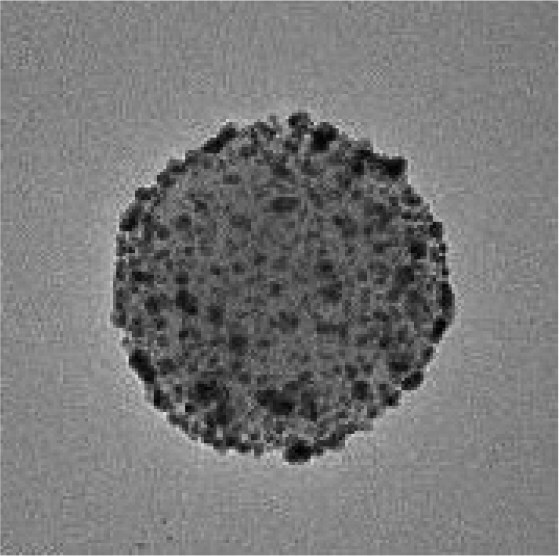	Scavenges ROS and promotes collagen production	TP	8 weeks	OA rats	Photothermal therapy enhances synergistic effects with good antimicrobial properties	[107]
	PdZn/Co_SA_-NC nanozymes	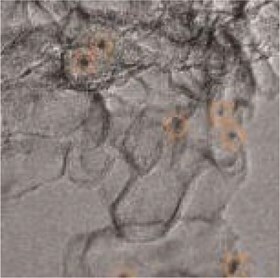	Modulation of the immune microenvironment	PdZn	8 weeks	ACLT rats	High catalytic activity and stability	[108]
	Mn_3_O_4_/UIO-TPP nanozyme	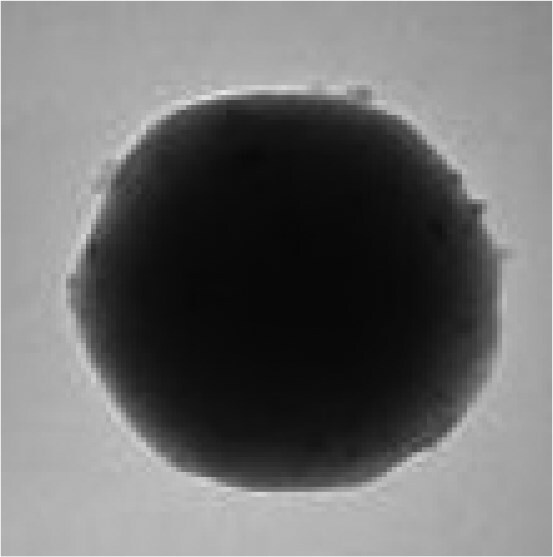	ROS clearance	Mn_3_O_4_	8 weeks	ACLT rats	Targeting mitochondria	[109]
Synthetic polymers	Acid-activatable polymeric curcumin NPs	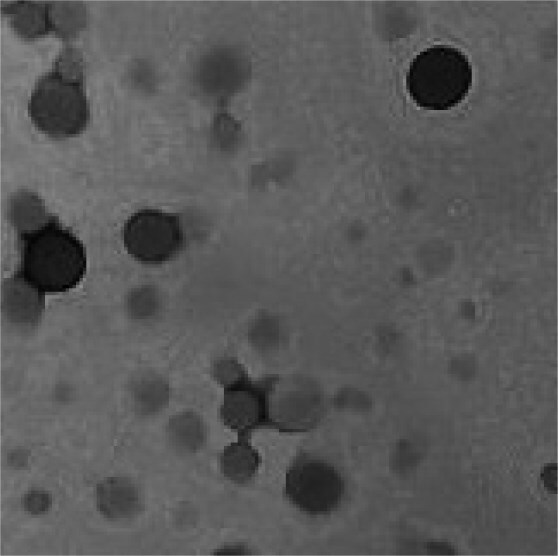	Inhibition of pro-inflammatory factor expression	Curcumin	7 d	MIA mice	Efficient, responsive release	[110]
Synthetic polymers	Rh-PLGA-NPs@NH_4_	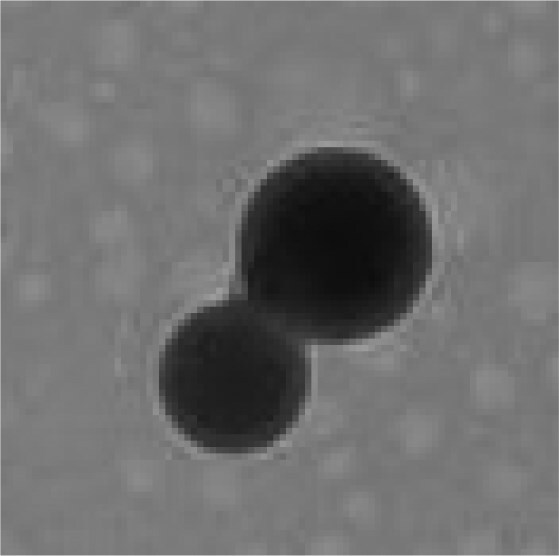	Inhibition of pro-inflammatory factor release, scavenging of ROS	Rh	7 d	–	pH-responsive release	[111]
	RNP	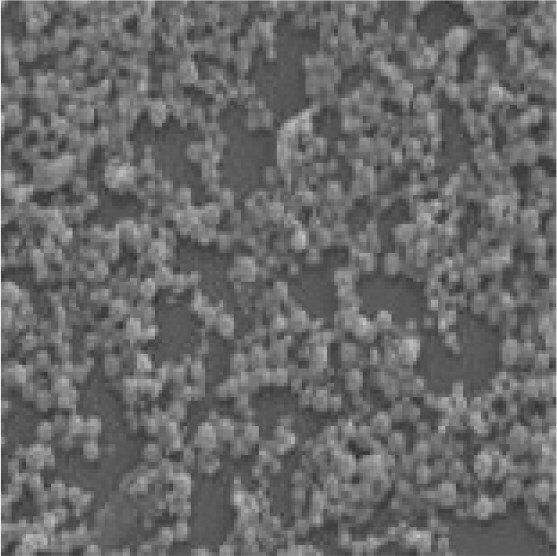	Prevention of chondrocyte senescence	Rapamycin	21 d	DMM mice	Maintains drug activity, low side effects, and slow release	[112]
	PLA/PEGDA-EDT@rGO-Fucoxanthin	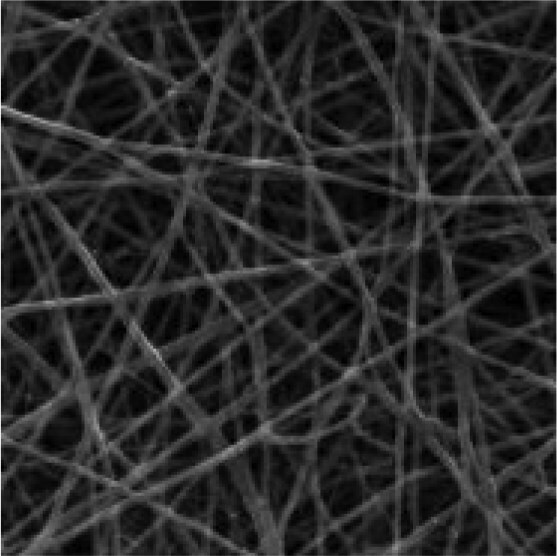	ROS scavenging, up-regulation of antioxidant enzymes	Fucoxanthin	8 weeks	ACLT rats	Smart ROS response, biodegradable	[113]
	NP@Poly	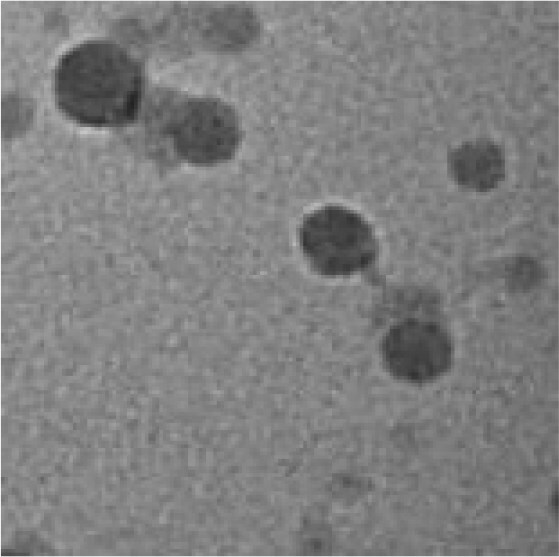	ROS clearance, macrophage repolarisation	Rapamycin	6 weeks	ACLT mice	Intelligent ROS response	[114]
Natural polymers	HA-Lipo-DIC/DEX	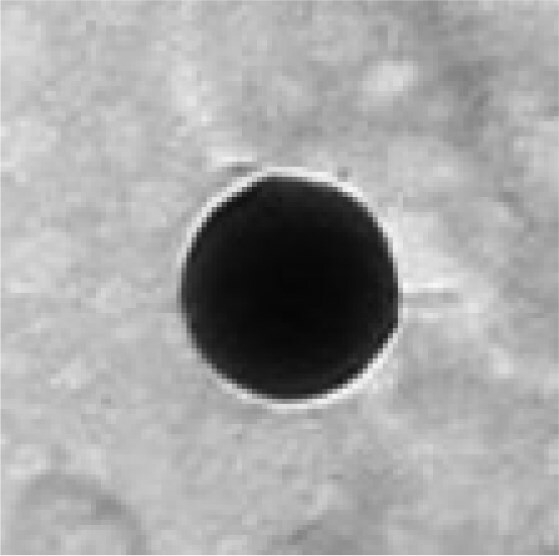	Analgesic and anti-inflammatory	DIC/DEX	4 weeks	FCA mice	Extended release time	[115]
	Celecoxib-loaded hyaluronan nanocapsules	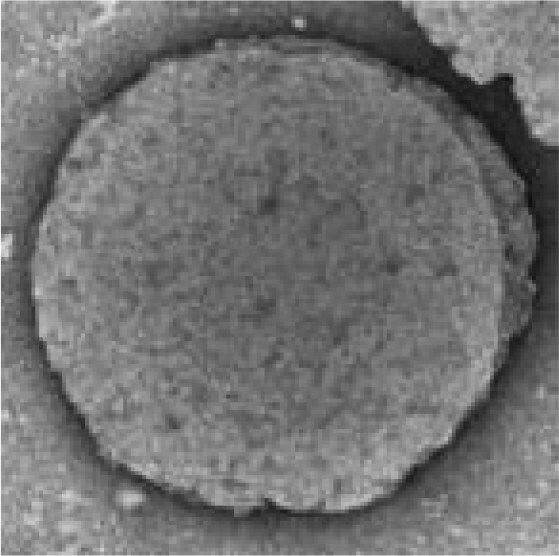	Analgesic and anti-inflammatory	Celecoxib	7 d	MIA rats	Extended release time, safe and non-toxic	[116]
	HA-αKG NPs	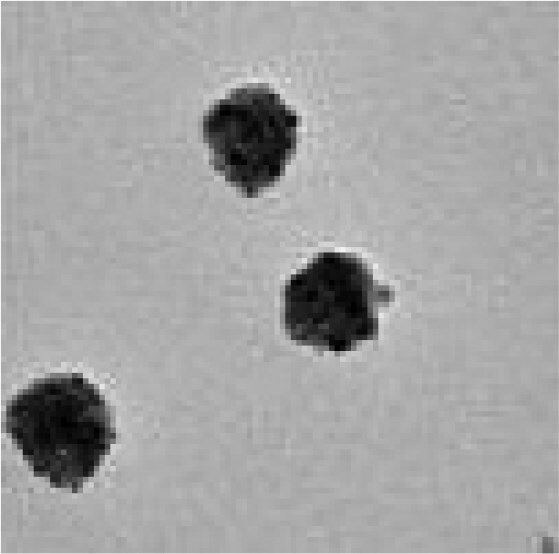	Analgesic, protects cartilage	αKG	2 weeks	DMM mice	pH-responsive release with high bioavailability	[117]
	HA hydrogel loaded with PL and CeO_2_-NPs	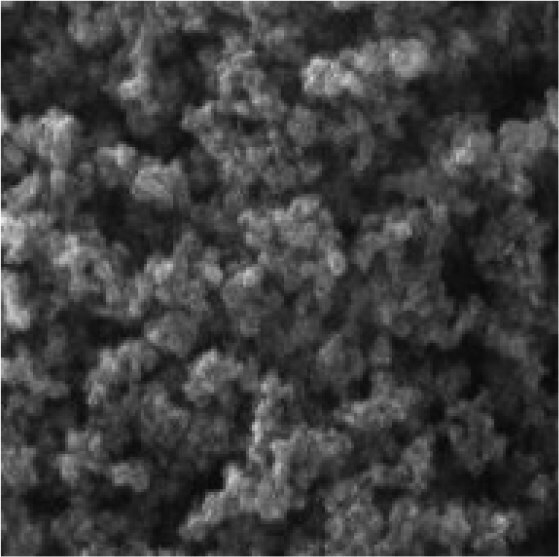	Inhibition of TNF-α synthesis	CeO_2_	72 h	–	Enhanced antioxidant effect, injectability	[118]
Natural polymers	GC/Fu@KAFAK NGs	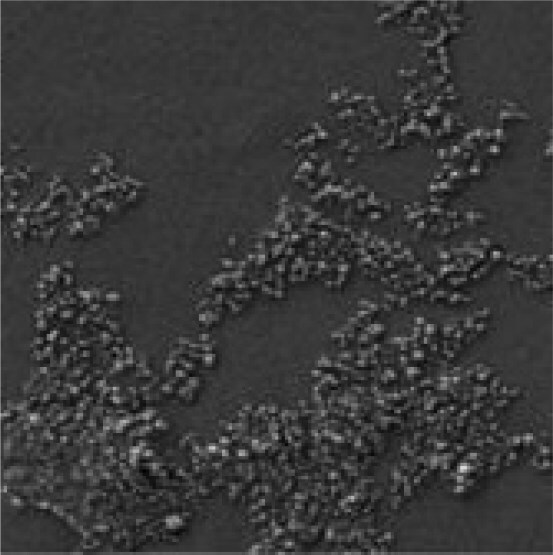	Down-regulation of IL-6 and TNF-α secretion levels and enhancement of chondrogenic marker expression	Fu@KAFAK	8 weeks	ACLT rats	Protects active ingredients and extends release time	[119]
	Hyt@tgel	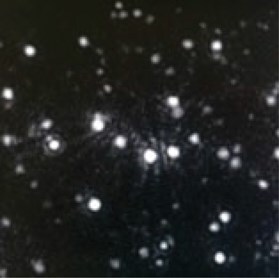	Inhibits the secretion of inflammatory factors	Hyt	14 d	–	Increased utilisation and longer release times	[120]

PDA: polydopamine; Hes: hesperidin; CAP: cartilage affinity peptide; ACLT: anterior cruciate ligament transection; DMM: medial meniscus surgical instability; FA: folic acid; Bai: baicalin; UIO: Zr-based organic framework; MIA: sodium iodoacetate-induced OA model; NIR: near-infrared; TP: tea polyphenols; PCN: zirconium-based porphyrin metal-organic framework; CoSA-NC: N-doped carbon-loaded Co single-atom; PdZn/NC: N-doped carbon-loaded PdZn ordered alloy; Rh: rhein; PLGA: polylactic acid–hydroxyacetic acid copolymer; rGO: reduced graphene oxide; Lipo: liposomal; DIC: diclofenac; DEX: dexamethasone; FCA: Freund's complete adjuvant; DIC: diclofenac; αKG: α-ketoglutarate; PL: platelet lysate; Fu: fucoidan; GC: glycol chitosan; Hyt@tgel: hydroxytyrosol-chitosan NPs.

#### Metal/metal oxide nanomaterials

**Figure 6.**
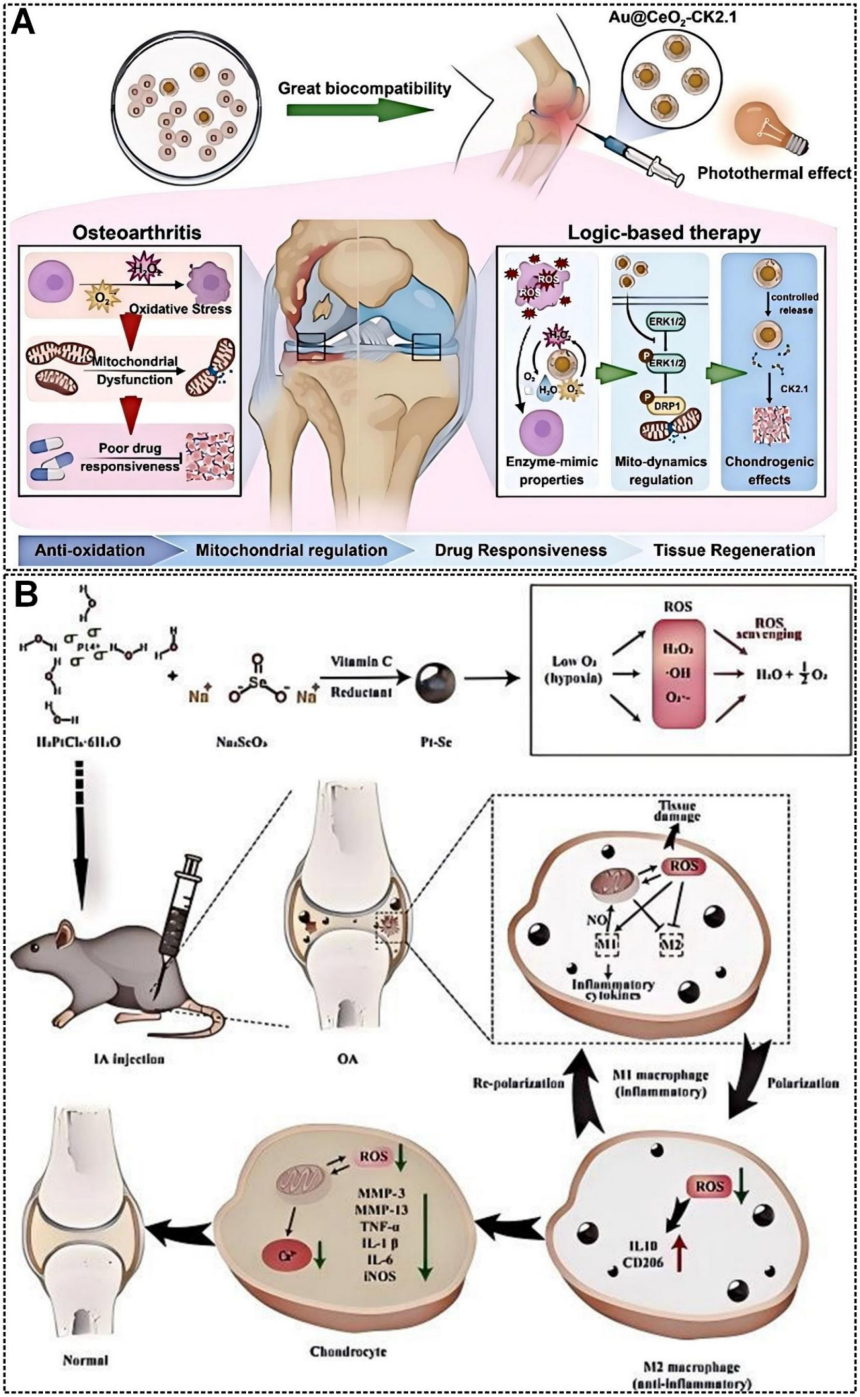
(**A**) Schematic diagram of the principle of Au@CeO_2_-CK2.1 for OA treatment. Reprinted with permission from Ref. [127]. (**B**) Schematic representation of Pt–Se nano-enzyme preparation and OA treatment. Reprinted with permission from Ref. [128].

Many inorganic NPs, such as cerium oxide (CeO_2_), manganese dioxide (MnO_2_) and platinum (Pt), have catalytic activities similar to those of antioxidant enzymes that can scavenge ROS. Compared with natural enzymes, these NPs are more stable and are widely used in OA therapy [121]. Metal/metal oxide NPs can enter cells via lattice proteins, lipid rafts and follicle-mediated endocytosis [122], and immune cells such as macrophages and neutrophils take up these NPs via phagocytosis [123]. For example, nanoceria has antioxidant activity and excellent therapeutic potential in high ROS environments. Dashnyam *et al.* applied different doses (100–2000 μg·mL^−1^) of nanoceria locally in a temporomandibular joint OA rat model and analysed tissue samples after 10 days of treatment. The results showed that nanoceria was effective in preserving the anatomical structure of articular cartilage and subchondral bone, especially at a dose of 500 μg·mL^−1^, which resulted in the preservation of cartilage hypertrophic layer and proteoglycans up to ∼80% (compared to only 30% in the OA group). Besides, nanoceria significantly reduced apoptosis, secretion of catabolic proteins (e.g., COX2/PGE2), and pro-inflammatory factors (e.g., IL-1β/TNF-α) and promoted the regenerative processes of M2-type macrophages polarized with (CD206/163) anti-inflammatory factors (IL-10) and chondrogenic proteins [124]. Similarly, CeO_2_ NPs were demonstrated to have the catalytic ability and to eliminate the excess ROS in OA, a property derived from the oxidation state of Ce^3+^ and Ce^4+^ on NP surfaces [125]. Gold NPs (AuNPs) can enhance the electron transport chain (ETC) activity of mitochondria to boost antioxidant capacity and exhibit excellent photothermal conversion efficiency, which allows AuNPs to enhance localized anti-inflammatory and antioxidant effects in photothermal therapy (PTT) [126]. Therefore, the researchers combined the advantages of these two NPs to design an egg yolk–shell nanostructure in which the core is AuNPs and the shell is CeO_2_ (Au@CeO_2_) (Figure 6A). These novel NPs ameliorate mitochondrial dysfunction in OA chondrocytes, restore chondrocyte mitochondrial homeostasis by eliminating excess ROS caused by inflammation and enhance cellular responses to external stimuli. Subsequently, effective cartilage repair and regeneration of OA was achieved by sequential release of the chondrogenic model drug peptide CK2.1 (Au@CeO_2_-CK2.1) and under laser irradiation [127]. Besides, PNPs also have enzyme-like catalytic properties to reduce LPS-induced ROS and pro-inflammatory cytokine expression, which has the potential to be applied in OA treatment [129]. However, the poor biocompatibility of PtNPs and simple aggregation of Pt particles may also lead to reduced catalytic activity. Therefore, the researchers tried to combine the essential trace element selenium (Se) with Pt to improve the stability and catalytic properties of NPs. Wei *et al.* synthesized Pt–Se composite NPs (Pt–Se NPs) by a simple chemical reduction method, which exerted a synergistic catalytic effect on ROS scavenging and macrophage repolarization in OA treatment (Figure 6B) [128].

#### Synthetic polymer nanomaterials

**Figure 7.**
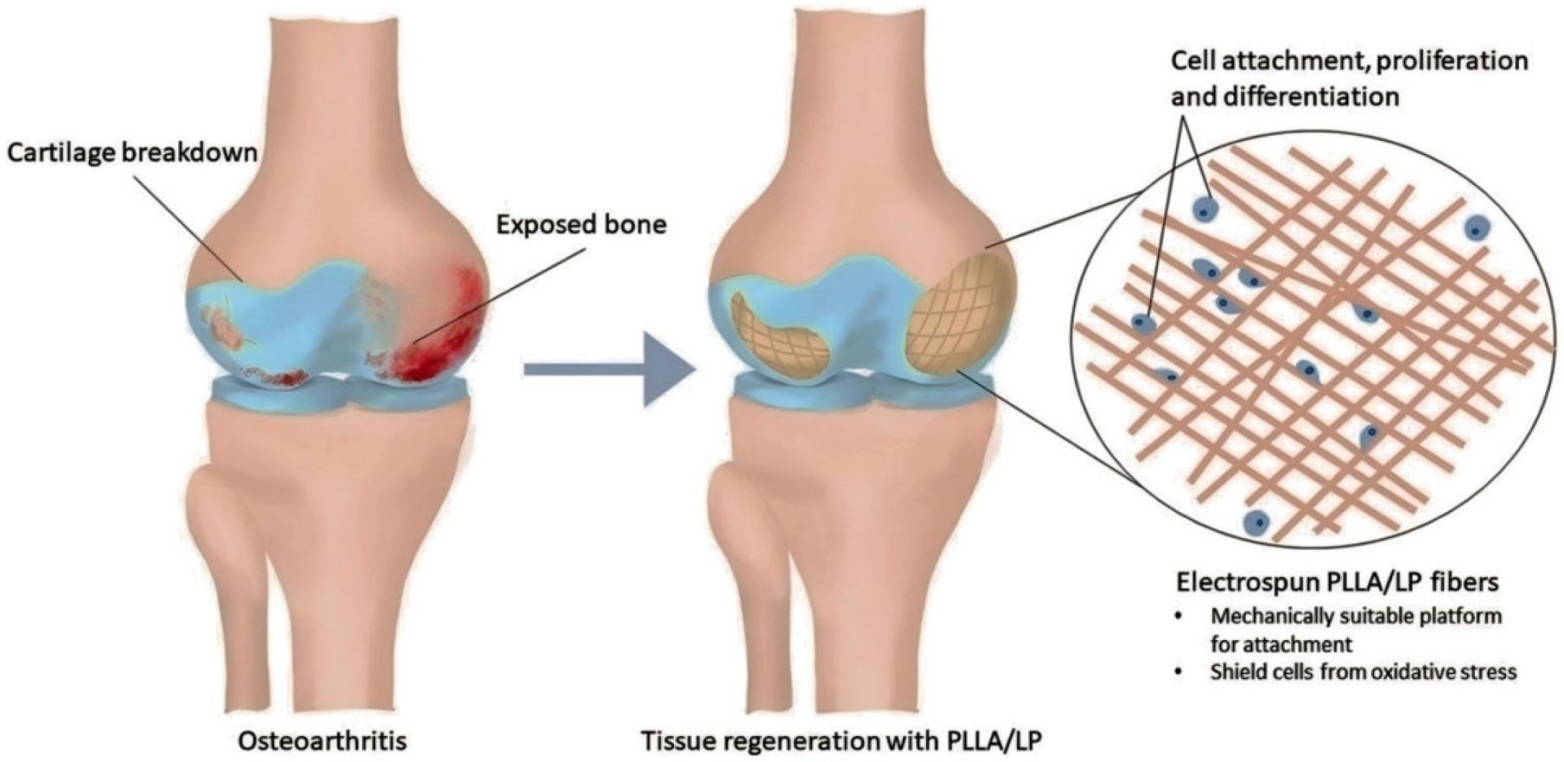
Schematic representation of PLA-lignin nanofibers for cartilage regeneration and OA treatment. Reprinted with permission from Ref. [134].

Synthetic polymer NPs have high stability and diverse structures, allowing for various functions. Polymer NPs can be doped with solid drug particles to function as drug carriers. The polymer and the drug are degraded *in vivo* through degradable chemical bonds to achieve controlled drug release in a specific environment. In addition, modification of particular targeting ligands on the surface of NPs can improve the precision of drug delivery [130]. Currently, lactic acid–hydroxyacetic acid copolymers (PLGA) nanomaterials have been widely studied and applied as drug carriers. PLGA is biodegradable, safe and low-toxicity. Therefore, Ma *et al.* designed a nanodrug carrier platform based on PLGA to encapsulate rapamycin (RNP) effectively. This platform can realize slow drug release and can enable the drug to be distributed in the joints, reducing the risk of systemic exposure to free drugs. The encapsulation efficiency of this PLGA drug carrier was as high as 85.2%, and the encapsulated rapamycin promoted chondrogenic differentiation and improved trauma-induced OA [112]. Polylactic acid (PLA) is an FDA-approved safe, biodegradable linear polyester that has been used in a wide range of medical applications such as fabricated bone implants, screws and sutures [131]. PLA has better biocompatibility and a prolonged release profile, improving solubility and increasing the local concentration of hydrophobic drugs [132]. Liu *et al.* attached adenosine molecules to poly(lactic acid)–poly(ethylene glycol) (PLA–PEG) nanofibers to prolong the half-life of adenosine in the joint. Animal experiments demonstrated that intra-articular injection of these nanofibers prevented the progression of traumatic OA in rats [133]. In contrast, Liang *et al.* grafted PLA onto lignin via ring-opening polymerization to improve the miscibility of lignin in nanofibers and enhance the mechanical properties (Figure 7). This hybrid nanofiber has good antioxidant activity and biocompatibility. The researchers prepared a lignin copolymer-based nanofiber cartilage repair scaffold by electrostatic spinning. The scaffolds can support MSCs and assist in chondrogenesis and cartilage repair, which has the potential for OA treatment [134].

#### Natural polymer nanomaterials

**Figure 8.**
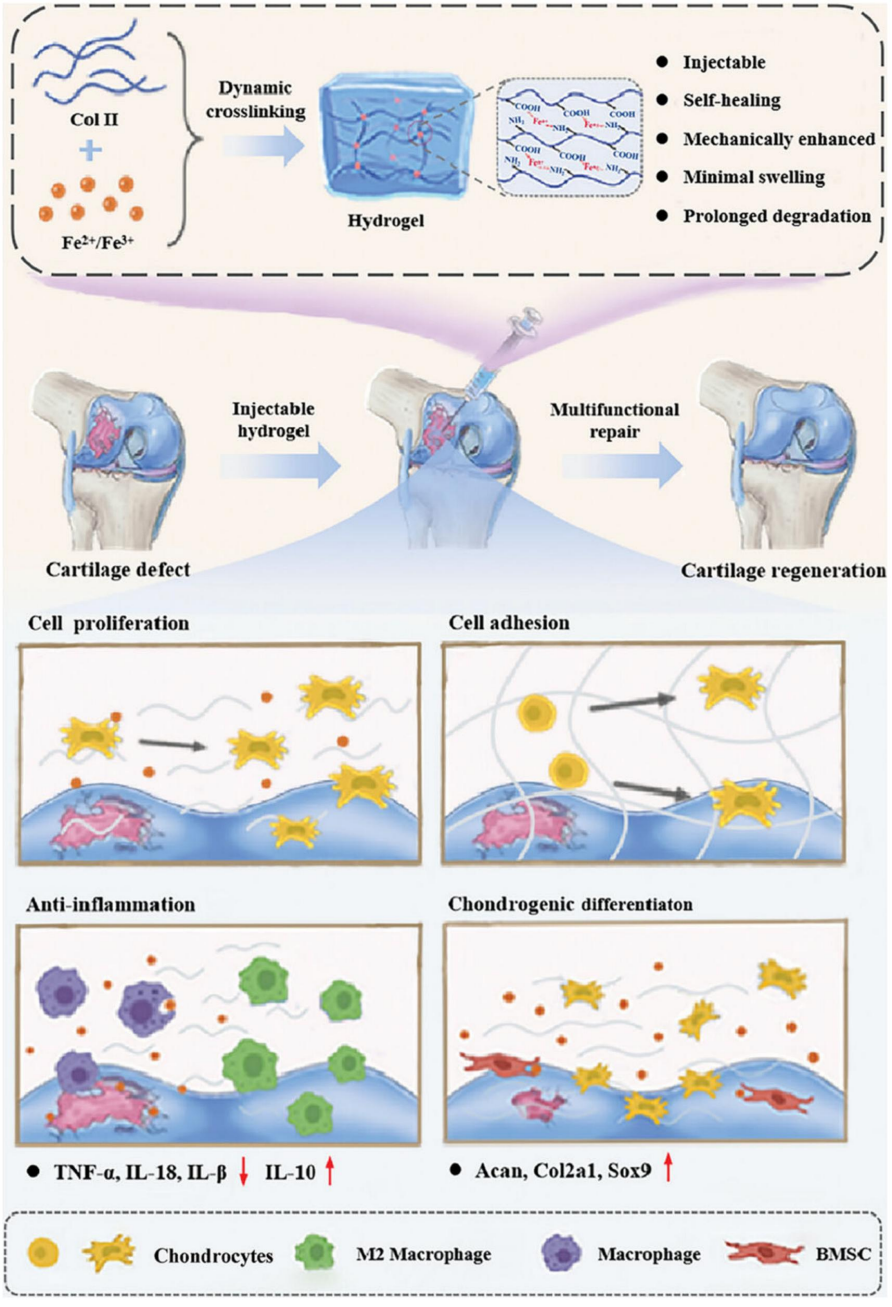
Schematic diagram of COL2 dynamically crosslinked with Fe^2+^/Fe^3+^ multifunctional hydrogel for OA treatment. Reprinted with permission from Ref. [137].

**Figure 9.**
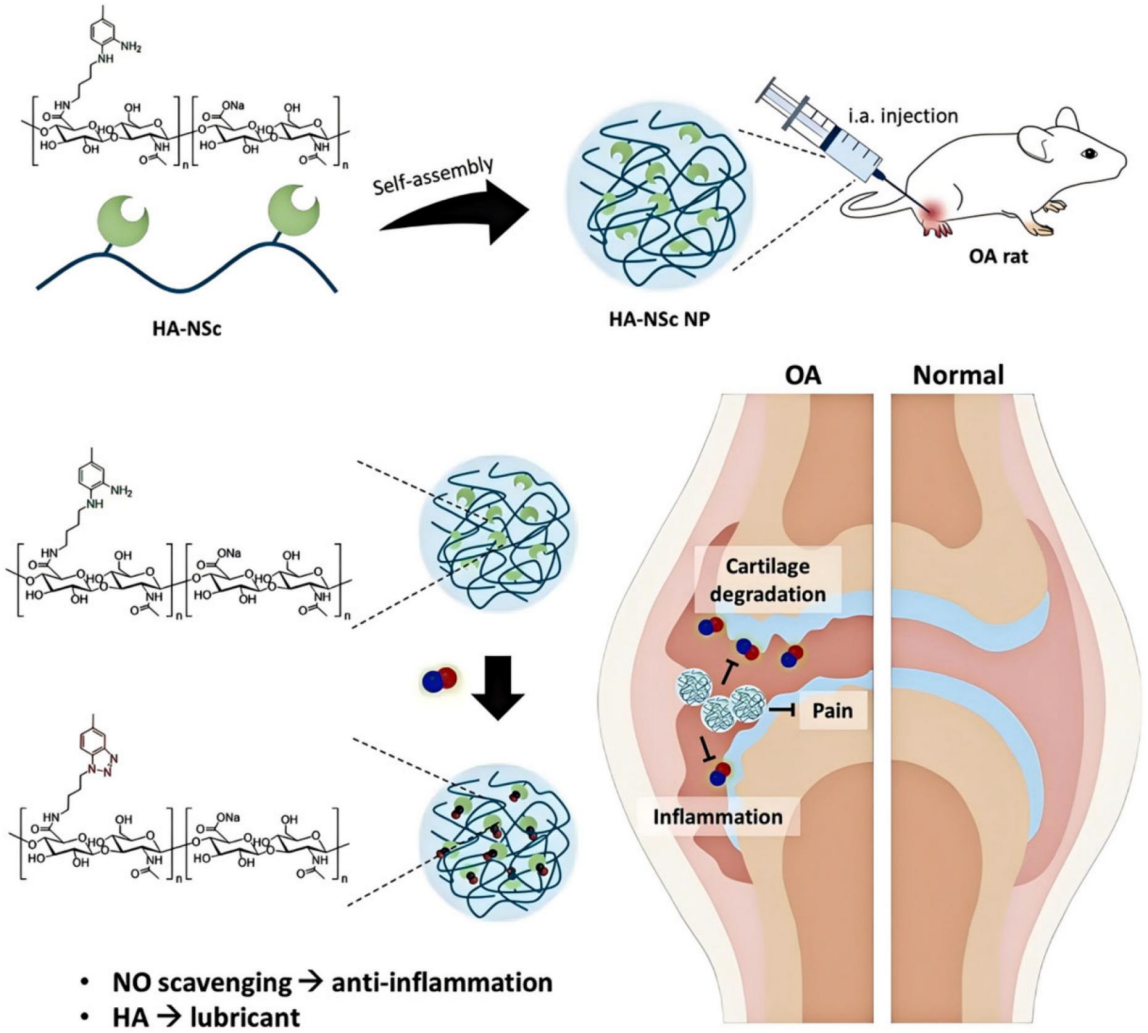
Self-assembled HA-NSc NPs for OA treatment. Reprinted with permission from Ref. [140].

Many NPs used for direct OA treatment consist primarily of natural polymers. These natural polymers in the joints, such as HA, CS and collagen, are present and are usually well-biocompatible, have low toxicity and can be smoothly degraded and metabolized in the human body [135]. For example, collagen, a major component of articular cartilage, contributes to structural maintenance, integrity and tissue homeostasis. COL2-based hydrogels have received attention in cartilage tissue engineering due to their good biocompatibility and bioactivity [136]. However, the difficulty separating COL2 and proteoglycans has led to the scarcity of COL2-based biomaterials and their limited mechanical strength, hindering OA treatment. Wang *et al.* found that Fe^2+^/Fe^3+^ efficiently and dynamically cross-links COL2, constituting a nanoporous 3D structure and forming an injectable self-repairing hydrogel with enhanced mechanical strength and degradation resistance (Figure 8). Fe^2+^/Fe^3+^ endows COL2 hydrogels with various properties, including accelerated cell proliferation, adhesion, chondrogenic differentiation and reduced inflammation. Experiments on articular cartilage defects in rats demonstrated the efficacy of multifunctional hydrogels in cartilage tissue rejuvenation and OA treatment [137]. HA, the main component of synovial fluid in the extracellular cartilage matrix, is a high-molecular-weight physical hydrogel that lubricates articular surfaces and reduces friction between articular cartilage and motion. It has good biocompatibility and is often used in OA treatment [138]. For example, Chatzaki *et al.* developed contrast agents ROS and hyaluronidase (HAse) as analytes. Since ROS and HAse trigger the OA coating cleavage on the surface of NPs, leading to the aggregation of AuNPs inside them, which enhances the CT signal, and enables the diagnosis of OA inflammation [139]. Based on the hydration, lubrication and joint friction reduction properties of HA, Nah *et al.* combined it with a novel NO scavenger, N1-(4-aminobutyl)-5-methylbenzene-1,2-diamine (NSc), to develop an NPs for OA treatment (Figure 9). These NPs retain the lubricating properties of HA and partially scavenge NO via NSc, reducing inflammation and cartilage degradation. Animal results showed that HA-NSc NPs prevented joint degeneration by maintaining structural stability, sustaining the lubricating properties of HA and decreasing the expression of MMP-1 and MMP-13 despite high NO conditions [140].

### Nanomaterial properties

#### Targeting

**Figure 10.**
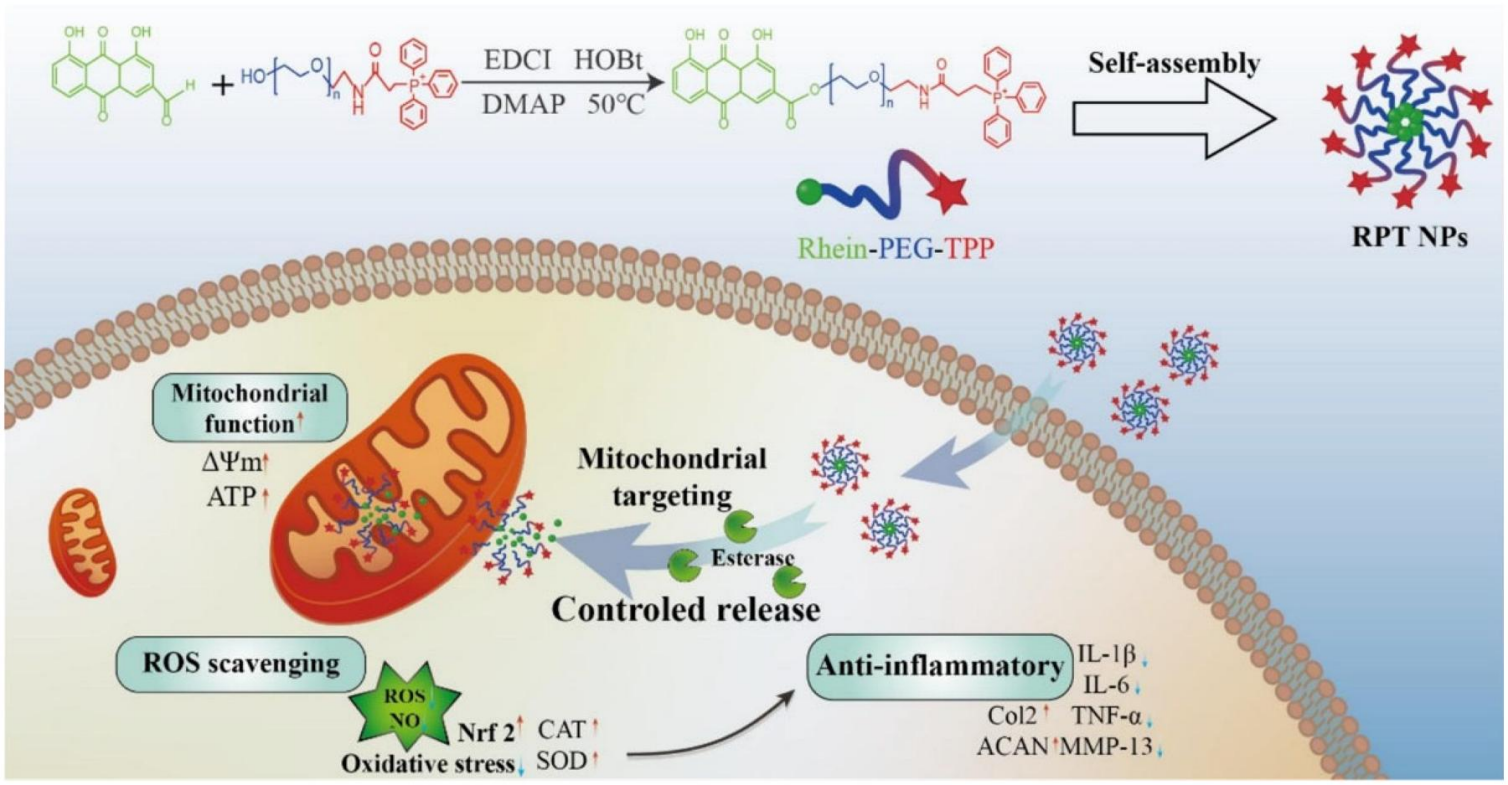
Schematic illustration of mitochondrial-targeting and esterase-responsive RPT NPs nano-prodrug for OA treatment. Reprinted with permission from Ref. [145].

Nanomaterials are materials with at least one dimension in the 1–100 nm range. This unique size range endows it with a large specific surface area and facilitates cellular uptake of NPs, thereby enabling passive targeting [141]. The NPs of a specific size are more likely to trigger cell membrane invagination and be endocytosed by the cell. Studies indicate that NPs of 50–100 nm can accumulate in OA-affected joints [142]. Besides, the size and shape of the nanomaterial can be optimized during the synthesis process. For instance, it can be designed into rod-shaped NPs that can more easily penetrate deep tissues [143]. The surface of nanomaterials can be modified by covalent modification and electrostatic adsorption [144]. For instance, Huang *et al.* synthesized an esterase-responsive, mitochondria-targeted nano-drug by modifying triphenylphosphine (TPP) molecules with PEG and covalently linking them to Rhein drug molecules. This nano-drug can self-assemble into NPs in water, target mitochondria and release drugs responsively to inhibit chondrocyte apoptosis [145]. This nanodrug delivery system effectively addresses limited drug solubility, insufficient *in vivo* stability and adverse gastrointestinal effects. It precisely delivers NPs to chondrocyte mitochondria via two-layer molecular modification, suppressing apoptosis and reducing inflammation (Figure 10). Collectively, custom-designed nanomaterials can target specific cells, tissues and organs. By attaching specific ligands to NPs, nanomaterials can recognize and bind to particular receptors. Moreover, nanomaterials can be engineered as responsive drug delivery systems capable of targeted release at OA-afflicted inflammatory sites through pH-triggered mechanisms, specific cytokine- or enzyme-activated responses and reactive oxygen species (ROS)-sensitive cascades. For example, Lan *et al.* developed a ‘smart’ dual-stimulus-responsive probe. It can efficiently and controllably deliver and release theranostic agents in response to high-MMP13 environments and acidic pH [146]. While Pan *et al.* developed a pH/redox-responsive nanogel as a carrier for geraniol, offering an effective OA delivery system. It can penetrate cartilage ECM, specifically bind to joints in the acidic OA microenvironment, prolong geraniol's residence in the joint cavity and enhance its efficacy. The experimental results indicate that nanogels possess excellent targeting capabilities, achieving high local concentrations in joints while reducing systemic exposure, minimizing the adverse effects of traditional drugs on the gastrointestinal system, liver, kidneys and cardiovascular system, and effectively protecting healthy tissues from damage [147].

#### Sustained-release capacity

**Figure 11.**
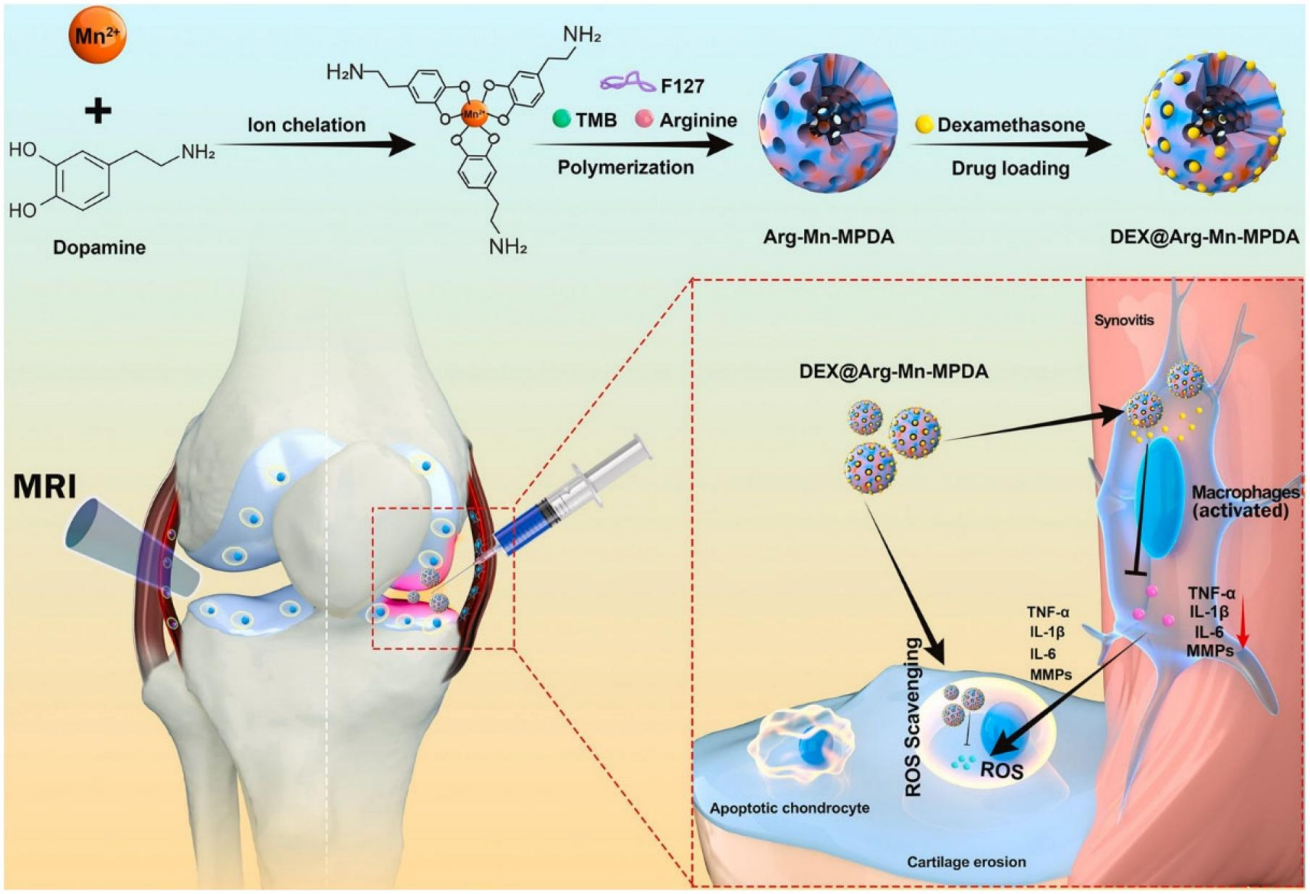
Schematic illustration of DAMM NPs for anti-inflammatory and antioxidant for OA treatment. Reprinted with permission from Ref. [148].

Nanomaterials have great potential in achieving precise and sustained drug release. Their degradation and drug diffusion rates can be custom-designed through kinetics-guided design. For example, precise control over the drug diffusion rate can be achieved by adjusting the material's cross-linking degree, such as the lactic/glycolic acid ratio in PLGA, or the pore structure, such as pore-size regulation in mesoporous silica (MSN). Liu *et al.* synthesized arginine and manganese-ion-doped mesoporous polydopamine NPs (DAMM NPs) loaded with DEX for OA intervention (Figure 11). The DAMM NPs' sustained DEX release can suppress synovial inflammation and inhibit TLR-3 production in chondrocytes, helping prevent chondrocyte apoptosis via the inflammatory-factor-dependent TLR-3/NF-κB signaling pathway [148]. Besides, their environment-responsive targeting ability ensures drug release precisely at the affected site. Coupled with a well-controlled release rate, this maximizes therapeutic efficacy. For example, Zhao *et al.* utilized the supramolecular interactions between azobenzene-modified MSN NPs (bMSN-AZO) and β-cyclodextrin-modified poly(2-methacryloyloxyethyl phosphorylcholine) (CD-PMPC) to construct visible-light-responsive, bifunctional, biodegradable MSN NPs for drug delivery and enhanced lubrication. Visible light efficiently triggers azobenzene isomerization, inducing drug release after penetrating the dermal tissue. The hydrated layer formed by CD-PMPC on the NPs' surface plays a crucial role in enhancing lubricity, which benefits OA treatment. Thus, by stimulating drug release via light irradiation that penetrates dermal tissue, drug release at the target site is achieved. And by up-regulating anabolic genes and down-regulating pro-inflammatory cytokines and catabolic genes, NPs exhibit remarkable anti-inflammatory effects [149]. In addition, He *et al.* designed pH-responsive MSN modified with polyacrylic acid (PAA) for pH-responsiveness. They loaded andrographolide (AG), which has anti-inflammatory and antioxidant properties but poor bioavailability, and the MSN improved its bioavailability. Experiments show that AG@MSNs-PAA has better anti-arthritis and chondroprotective effects than AG, as evidenced by lower expression of inflammatory factors and better prevention of proteoglycan loss. Thus, the AG@MSNs-PAA nanoplatform can be developed as a promising OA-specific and controlled-release drug delivery system [150].

#### Properties of different types of nanomaterials

In addition to the above-mentioned common advantages, different nanomaterials have respective advantages in OA treatment due to their differing properties. Inorganic NPs are made of inorganic materials. They have controllable structures and unique surface chemistry. They can release metal ions in the body, acting as therapeutic agents [151]. Metallic NPs, with good stability and a long half-life, are effective drug carriers. Some even have unique therapeutic effects [152]. For example, AuNPs have antioxidant and anti-inflammatory properties. Chen *et al.* used hyaluronic acid (HA) to modify AuNPs (HAuNPs), enhancing biocompatibility and therapeutic potential. HAuNPs target degenerative cartilage and treat early-stage knee OA. *In vitro*, they do not affect chondrocyte viability or apoptosis rates, and protect the chondrocyte phenotype from IL-1β stimulation. After intra-articular injection, they accumulate in injured cartilage, suppress MMP13 expression and prevent further cartilage degradation [153].

**Figure 12.**
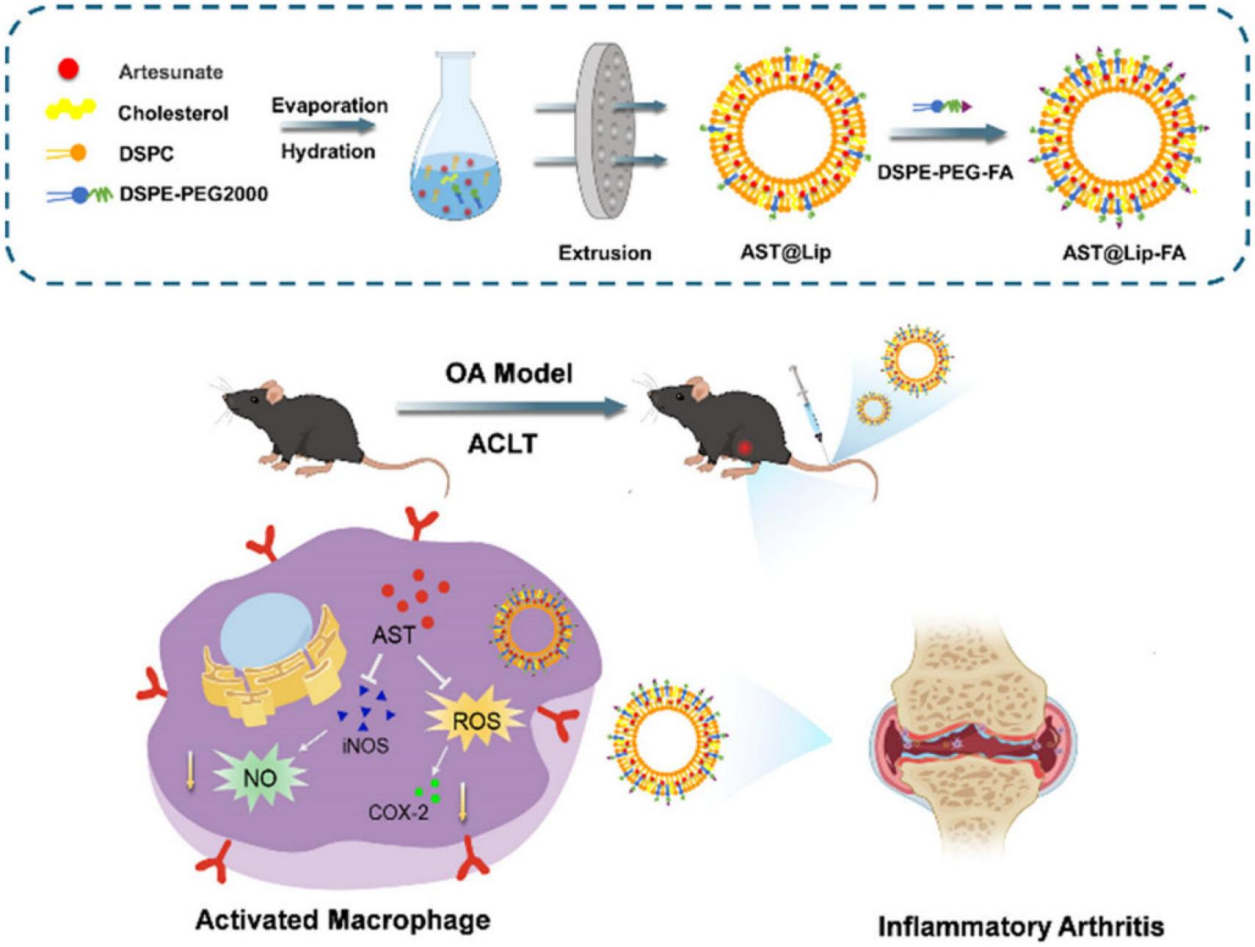
Preparation process of AST@LIP-FA and the treatment of OA. Reprinted with permission from Ref. [161].

Polymeric nanomaterials, composed of natural or synthetic polymers, can be custom-designed for specific structures, especially for drug-delivery-related surface modification. Most of them also have good biocompatibility and biodegradability [154]. Drugs can be encapsulated within polymeric nanomaterials or conjugated to their surface via covalent bonds [112]. Synthetic polymers can carry large amounts of drugs and can be designed to deliver hydrophobic or hydrophilic drugs depending on their composition and structure. However, their stability and safety need careful validation [155]. Natural polymers such as chitosan and alginate are usually more biocompatible and biodegradable and are often used in OA therapy. For example, Nabizadeh *et al.* incorporated chitosan NPs (CS NPs) loaded with fisetin and the chondroprotective agent kartogenin (KGN) into HA oxidized to generate aldehyde groups, which were then cross-linked with adipic dihydrazide (ADH) to form a hydrogel. *In vitro* evaluations show that this drug-loaded NP hydrogel system can treat OA effectively by inhibiting inflammation quickly and supporting cartilage regeneration [156]. Nanogels (NGs) have good stability and can respond to stimuli like pH, MMP [157] and electric potential [158]. This allows them to reach OA-damaged areas and release drugs sustainably over a long period. For example, Sun *et al.* constructed an NG by functionalizing the surface of zeolitic imidazole-framework-8 loaded with kartogenin (KZIF@HA) via an amide reaction. The inherent hydrophilicity of HA enabled KZIF@HA to form NGs spontaneously, prolonging drug release in the OA microenvironment. Experiments showed that KZIF@HA released drugs sustainably for a month. Compared to KZIF, it has a lower risk of synovial fluid leakage, better cartilage penetration and is reparative to chondrocytes [159]. Nanoliposomes are spherical structures made of phospholipid bilayers with internal drug-loading spaces. They are ideal for delivering lipophilic drugs, can fuse with cell membranes, have good biocompatibility and can enrich immunocytes in OA treatment [160]. Nanoliposomes are also sensitive to external stimuli such as shear force, temperature and pH. Zhao *et al.* developed folate-functionalized astaxanthin-loaded liposomal NPs (AST@LIP-FA) leveraging nanoliposomes' characteristics (Figure 12). The liposomes enhanced AST's solubility and stability, while folate targeted macrophages, boosting cellular uptake and delivering AST to OA sites. Experiments showed AST@LIP-FA effectively scavenged intracellular ROS/RONS. In ACLT-induced OA mice, AST@LIP-FA precisely accumulated in inflamed joints, had long-term retention and fully utilized AST's anti-inflammatory, antioxidant and chondroprotective effects to effectively mitigate OA progression [161].

### Nanomaterials for OA treatment

#### Nanomaterials as drug carriers

**Figure 13.**
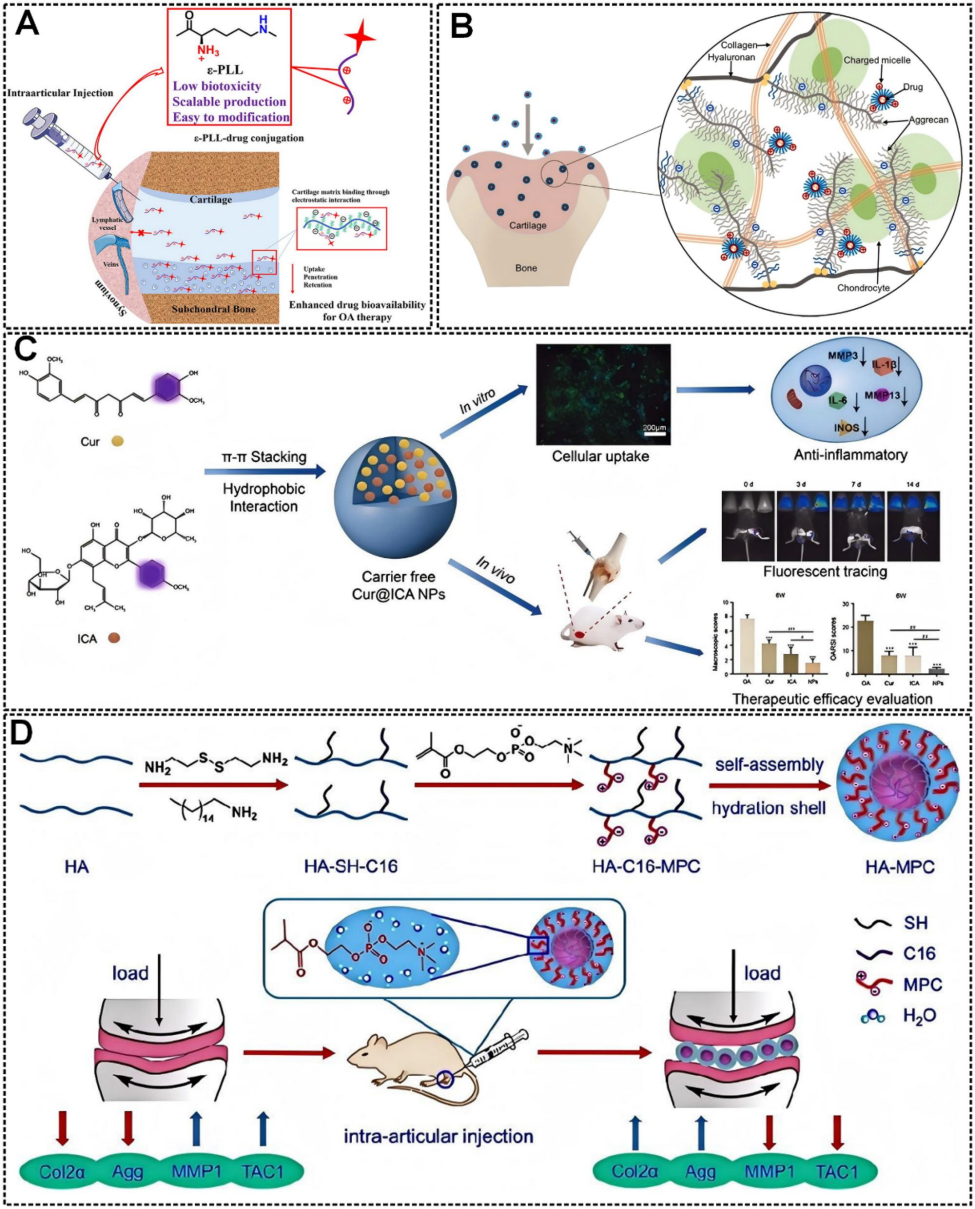
(**A**) Schematic representation of ε-PLL as a cationic small molecule carrier for drug delivery to cartilage. Reprinted with permission from Ref. [165]. (**B**) Electrostatic interactions between cationic micelles and negatively charged cartilage aggregates are illustrated. Reprinted with permission from Ref. [166]. (**C**) The preparation process for carrier-free Cur@ICA NPs with synergistic therapeutic OA. Reprinted with permission from Ref. [167]. (**D**) Schematic representation of lubricated HA-MPC nanospheres developed by amidation (HA-SH-C16), mercapto-alkene click (HA-C16-MPC) and self-assembly (HA-MPC) synthesis for OA treatment. Reprinted with permission from Ref. [168].

The utilization of nanomaterials as drug carriers presents multiple advantages. For example, NPs can prevent the rapid systemic distribution of small-molecule drugs through bloodstream circulation, thereby mitigating associated toxic side effects. Besides, nanomaterials exhibit a substantial specific surface area, enabling precise targeting capabilities through surface functionalization modifications. NPs have an ideal drug loading to prolong circulation time in the body and optimize therapeutic efficacy. Currently, many groups have achieved targeted transportation and slow controlled release of drugs, and better efficacy in OA therapy by surface modification of drug carrier NPs [162]. Drug clearance in works using intra-articular (IA) drug delivery interventions for OA treatment is high, and the delivery process is limited by the highly negatively charged ECM and the dense cartilage [163]. Nanodelivery systems can also extend drug retention time in the joint, improve ECM penetration and enable targeted delivery. For example, Lina *et al.* to address the problem of IA injections facing rapid drug clearance and producing off-target effects, developed a novel inclusion of poly(lactic acid–hydroxyacetic acid copolymer) (PLGA) nanocomposites—4-arm-poly (ethylene glycol)-maleimide (PEG-4MAL) microgel NPs to load binding peptides that specifically bind to chondrocytes or synovial cells. Finally, they successfully encapsulated the model drug rhodamine B with a gel synthesized by microfluidics and achieved a near-zero level kinetic drug release *in vitro* for 16 days. *In vitro* experiments, PEG-4MAL microgels exhibited specific binding ability to rabbit and human synoviocytes and bovine articular cartilage. In a rat post-traumatic knee OA model, this microgel was retained in the joint cavity for at least three weeks without inducing degenerative joint changes [164]. Besides, Lin *et al.* developed an ε-poly-L-lysine (ε-PLL) scaffold for OA therapeutic drug delivery. The ε-PLL is a biodegradable polyamino acid nanocarrier formed by lysine condensation to less than 10 nm. These carriers are synthesized by covalent bonding between α-carboxy and ε-amino groups, and the exposed α-amino group is protonated at physiological pH, conferring a cationic charge density to the nanocarriers and facilitating reversible binding to the cartilage matrix. The results showed that more than 60% of the 4.6 kDa ε-PLL can be retained within the soft of fresh porcine cartilage explants for 10 days. Compared to poly(ethyleneimine) (PEI) and poly(amidoamine) (PAMAM) dendritic cationic polymers, ε-PLL had a higher penetration into the cartilage matrix, which may be attributed to its spatial site-blocking effect. Furthermore, ε-PLL did not show toxicity to human chondrocytes and MSCs (hBMSC), whereas PEI and PAMAM dendritic polymers significantly affected cell viability. The ε-PLL drug delivery scaffold has effective drug-delivery capability, good biocompatibility, high intrachondral retention and penetration and cartilage protection properties in OA treatment (Figure 13A). Besides, ε-PLL functions as a safe and effective cationic macromolecular carrier that achieves deep tissue penetration and sustained retention in cartilage through reversible electrostatic interactions with the negatively charged ECM [165]. Zhu *et al.* recently synthesized triblock copolymers NPs (PDMAEMA–PCL–PDMAEMA) consisting of cationic poly(N,N-dimethylaminoethyl methacrylate) (PDMAEMA) and poly(ε-caprolactone) (PCL) with dimensions of 20–30 nm by the nanoprecipitation technique. The experiments showed that the permeability and retention of cationic micelles with positive charge in cartilage were significantly improved. The PDMAEMA–PCL–PDMAEMA NPs loaded with free model drugs could penetrate the cartilage layer, remain in the cartilage for at least 4 days and overcome the obstacles to drug delivery by cartilage-like negatively charged tissues (Figure 13B). This nanomaterial's functionality is mediated by electrostatic interactions arising from charge complementarity between its cationic surface and the anionic components of the cartilaginous matrix, enabling deep tissue penetration for targeted therapeutic delivery [166].

#### Nanomaterials for direct OA treatment

Besides OA drug nanocarriers, many nanomaterials can be used directly in OA treatment and are referred to as therapeutic nanomaterials. Therapeutic nanomaterials have excellent targeting and membrane penetration properties, which can reduce the risks associated with high drug concentrations in plasma and are gradually being recognized in the medical field [169]. Meanwhile, surface modification can make nanomaterials photosensitive, thermosensitive and magnetic, and external stimuli can be used to make these particles respond to the patient to achieve therapeutic effects [170]. Besides, NPs with direct therapeutic effects on OA usually contain elements naturally occurring in the joints, such as HA, decellularized matrix, collagen, etc., and have good biocompatibility, biodegradability and low toxicity. Currently, medications used to treat OA such as analgesics, non-steroidal anti-inflammatory drugs, HA and corticosteroids are used to alleviate symptoms rather than to alter the course of the disease. Reactive oxygen species (ROS) and nitrogen oxides (RNS) play a key role in OA development. In OA patients, antioxidant mechanisms are dysregulated, failing to effectively scavenge ROS and RNS leading to increased oxidative stress, causing DNA, lipid and protein damage. Zhong *et al.* prepared melanin NPs as a novel free radical scavenger for OA treatment. Melanin NPs with a large size and the ability to be retained in the joints for a longer period facilitate sustained ROS/RNS inhibition and OA management. Besides, melanin NPs have an antioxidant mechanism and protect mice from γ-radiation-induced DNA damage [171]. Clinical applications are limited by the rapid clearance of many drugs in the joint cavity. Although NP-based medications have been developed as carriers, they may trigger side effects or toxicity. Dai *et al.* designed a novel carrier-free self-assembled nanomedicine, curcumin (Cur)/icariin (ICA) NPs, with tunable particle size through π–π stacking interactions of two natural small molecules, providing a structural basis for engineering stable nanopharmaceuticals through precisely orchestrated non-covalent interactions (Figure 13C). The Cur/ICA NPs have low cytotoxicity, high cellular uptake and sustained drug release, effectively inhibiting inflammatory cytokines and reducing cartilage degeneration. *In vitro* and *in vivo* experiments, the Cur/ICA NPs exhibit synergistic anti-inflammatory and chondroprotective effects superior to those of Cur or ICA alone. Interestingly, the NPs could self-monitor their retention in the joints by autofluorescence [167]. OA causes irreversible damage to cartilage due to increased mechanical friction in the joints, so restoration of joint lubrication is essential for treatment. Zheng *et al.* synthesized HA-based amphiphilic ion nanospheres (HA-MPC) with choline phosphate groups on their surfaces, significantly enhancing the lubrication effect through a hydrated lubrication mechanism (Figure 13D). The fabrication protocol initiates with thiol-functionalization of HA via amidation with hexadecylamine, followed by MPC conjugation through thiol-ene click chemistry. This molecular engineering creates amphiphilic derivatives that undergo entropy-driven self-organization into nanospheres via hydrophobic collapse, ultimately forming an intra-articular injectable therapeutic platform with controlled biodistribution characteristics. The friction test showed that HA-MPC nanospheres reduced the coefficient of friction by 40% compared to HA. The results showed excellent biocompatibility, up-regulation of cartilage anabolic genes and down-regulation of catabolic proteases and pain-related genes [168].

#### Nanomaterials for stem cell-based OA therapy

**Figure 14.**
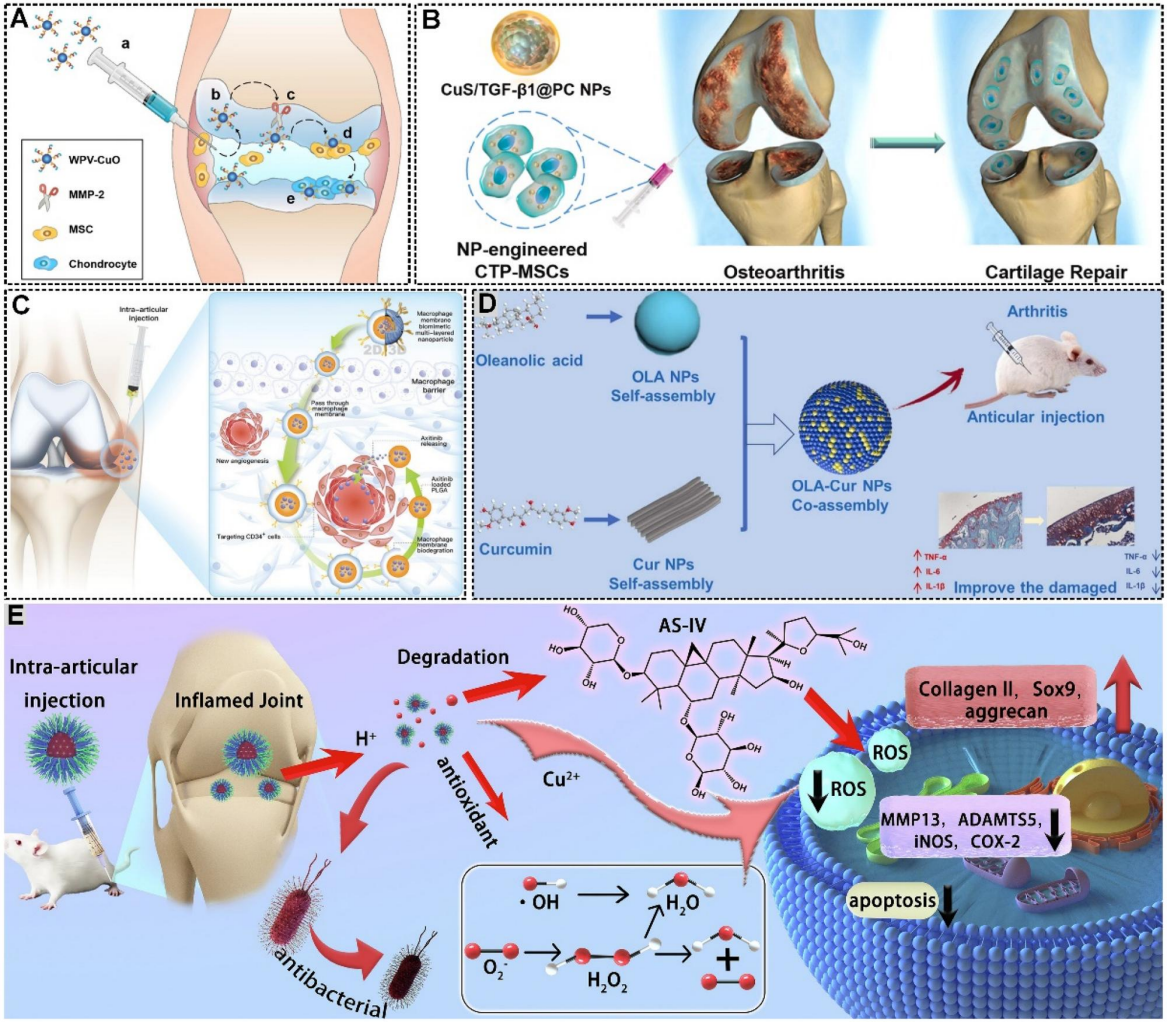
(**A**) Schematic illustration of the application of WPV-CuO NPs in OA therapy. Reprinted with permission from Ref. [176]. (**B**) Schematic representation of OA therapeutic CuS/TGF-β1 NPs based on MSCs. Reprinted with permission from Ref. [177]. (**C**) Schematic of macrophage membrane-biomimetic multi-layered nanoparticles alleviates OA progression by inhibiting synovium angiogenesis. Reprinted with permission from Ref. [178]. (**D**) Schematic diagram of supramolecular co-assembly strategy to construct OLA-Cur co-assembled composite nano-mitigation system for the treatment of OA. Reprinted with permission from Ref. [179]. (**E**) Diagram illustrating the CSP@AS-IV nanomedicine for OA therapy. Reprinted with permission from Ref. [180].

In recent years, stem cell therapy, emerging as a promising therapeutic approach, has shown great potential OA treatment field. MSCs are mesoderm-derived cells with self-renewal capacity, found in bone marrow, adipose tissue, synovium, etc., and are widely used for OA therapy [172, 173]. MSCs can differentiate into osteoblasts and chondrocytes, and promote tissue regeneration by secreting bioactive substances like EVs, immunomodulatory factors and cytokines [174, 175]. However, due to the inflammatory microenvironment in the joints of OA patients, there are still problems in clinical applications, such as insufficient survival rate of MSCs after injection and low directed differentiation efficiency. Nanomaterials can achieve controlled and targeted cell recruitment, provide a suitable microenvironment for cell survival and enable fluorescence imaging to track cell differentiation and proliferation. Thus, they show potential in stem cell-based OA therapy. For example, Lu *et al.* selected CuO-based NPs, which can promote chondrogenesis of MSCs and enhance cartilage formation (Figure 14A). They utilized a COL2 and MSC dual-targeted functional peptide (WPV) for cartilage penetration and MSC recruitment, inserting a matrix metallopeptidase 2 (MMP-2)-sensitive sequence as a spacer to respond to the OA microenvironment. In rat experiments, peptide-guided NPs actively target cartilage, are broken down by abundant MMP-2 in the OA microenvironment and expose the internal MSC-targeting peptide for MSC recruitment. After recruitment, the MSCs are induced by CuO NPs to undergo chondrogenesis, achieving effective OA treatment through cartilage regeneration [176]. Dong *et al.*, on the other hand, utilized NP to ameliorate the effects of the OA inflammatory microenvironment on MSCs. They designed a biomimetic NP targeting M1 macrophages to deliver itaconic acid (ITA), inhibiting M1 macrophage pyroptosis and reducing inflammation. This biomimetic NP, coated with folic acid-modified cell membranes and loaded with ITA, can target lesion areas, reducing drug side effects on normal tissues and increasing drug concentration in pathological tissues. In an OA rat model, intra-articular injection of these NPs can protect articular cartilage from OA progression and reduce inflammation [181].

Nanomaterials combined with gene editing or plasmid transfection techniques can promote chondrogenesis of stem cells. These technologies show great promise in promoting the directed differentiation of stem cells into chondrocytes. For example, Cai *et al.* designed CuS NPs and modified them with 3-aminopropyltriethoxysilane to obtain CuS–NH_2_, which promotes binding to negatively charged plasmid DNA encoding TGF-β1. In addition, they coated CuS/TGF-β1 NPs with phosphatidylcholine (PC), enhancing biosafety and enabling effective pDNA transfer into MSCs, thus forming CTP-MSCs (Figure 14B). These NPs-enhanced MSCs can promote chondrogenesis and inhibit ECM degradation. Intra-articular injection of CTP-MSCs in OA mouse models significantly promotes cartilage repair and successfully treats OA [177]. SOX9, a key transcription factor for chondrogenesis, binds to the chondrocyte-specific COL2A1 enhancer sequence and activates COL2A production by MSCs [182]. Thus, Wu *et al.* designed CuO-based NPs, loading them with plasmid DNA encoding Sox9 and recombinant bone morphogenetic protein 7 (BMP7), which inhibits hypertrophic differentiation during chondrogenesis and promotes cartilage matrix synthesis. Once taken up by MSCs, the NPs are retained for over 14 days. The SOX9 plasmid is rapidly transcribed, promoting chondrogenesis. These NPs have shown efficient chondrogenic repair in surgically induced OA mouse models and damaged cartilage samples from OA patients [183].

### Effects of nanomaterials on the immune response

The early stages of OA pathogenesis are primarily influenced by factors such as external injury or disruption of biomechanical homeostasis, leading to inflammation induced by the immune system [135]. For example, sensitive to mechanical stress, chondrocytes and synoviocytes produce hydrolytic enzymes such as MMP13 that recruit immune cells. Therefore, immune cells, such as B cells, T cells and macrophages, infiltrate the inner synovium during OA pathology, leading to alterations in catabolic substances and cytokine production that promote inflammation. Injecting nanomaterials into diseased joints releases the loaded drug to act on immune cells and suppress inflammation.

#### Effects of nanomaterials on immune cells

Macrophages play a crucial role throughout the progression of OA, with the M1 pro-inflammatory phenotype releasing pro-inflammatory cytokines while the M2 phenotype releases anti-inflammatory factors. KAFAK is an anti-inflammatory cell-penetrating peptide. Besides, as a pro-inflammatory factor, leptin mediates inflammatory signaling through the leptin receptor (LEPR), and inhibition of LEPR signaling attenuates the negative effects of high leptin concentrations. Based on this, Zhou *et al.* developed biomimetic NPs (M2H@RPK) encapsulating M2 macrophage membranes (M2M), combining iRGD, KAFAK and shRNA-LEPR, and modified by HA to make M2 membranes negatively charged. The system targets the inflamed synovium, blocking the inflammatory cascade and promoting environmental repair. *In vitro* and *in vivo*, the M2H@RPK has good cellular uptake efficiency, macrophage repolarization, anti-inflammatory and chondroprotective effects, showing the potential for palliative OA treatment [184]. One feature of OA is that angiogenesis mediates synovitis, so anti-angiogenesis is considered an effective OA therapy [185]. Macrophages in the synovial lining layer form a ‘protective barrier’ that blocks communication between the synovial joint cavity and the sublining layer. Some studies have identified CD34 synovial cells as ‘sentinel cells’ for synovial angiogenesis. Therefore, Liao *et al.* constructed a biomimetic multilayer NPs by combining PLGA loaded with axitinib (Atb) and macrophage membranes modified with CD34 antibodies (Atb@NP@Raw@CD34) (Figure 14C). This formulation can specifically deliver Atb to the sub-lining layer, maintaining anti-angiogenic effects without immunoeLimi nation. This design enables the NPs to penetrate the macrophage barrier, achieve specific targeting of CD34 cells, avoid macrophage degradation and effectively suppress angiogenesis [178]. Currently, most nanomaterials targeting immune cells primarily affect macrophage behavior, especially their polarization. However, future nanomaterials that target other immune cells like T cells and B cells, or act on multiple immune cells jointly, will be among the most promising research directions for OA treatment.

#### Effects of nanomaterials on immune cytokines

In addition to targeting immune cells, nanomaterials can also treat OA by acting on cytokines. IL-1β, a key pro-inflammatory cytokine, is recognized as a potential therapeutic target for OA. While the IL-1Ra can block the IL-1β signaling pathway, its suboptimal efficacy in clinical trials may be attributed to structural barriers posed by the chondrocyte ECM and rapid drug metabolism. Conventional single anti-inflammatory strategies have limited efficacy in severe cartilage degradation, hence the need to introduce cartilage repair. Although IL-1β inhibitors and cartilage repair agents are used, their short half-life and adverse effects limit their application. To address these issues, Wang *et al.* developed nanobiotics that formed self-assembled nanobiotics (ICN) by noncovalently assembling IL-1Ra and chondroitin sulfate. The nanomedicine showed excellent biocompatibility, low immunogenicity, high biodegradability, strong anti-inflammatory and extraordinary cartilage repair efficacy. ICN significantly inhibited IL-1β-induced chondrocyte apoptosis in OA rats, promoted COL2 expression and effectively alleviated pain symptoms. Besides, ICN significantly promoted the expression of Col II and GAGs and down-regulated inflammatory factors in the cartilage matrix through synergistic anti-inflammatory and cartilage repair properties [186]. LPS may induce the release of pro-inflammatory cytokines such as TNF-α, IL-6 and IL-1β. To target these immune cytokines, Liu *et al.* selected oleanolic acid (OLA), a natural small molecule with self-assembling properties, to co-assemble with Cur to form composite NPs. Cur can inhibit the release of TNF-α, IL-6 and IL-1β, and boost the secretion of the anti-inflammatory cytokine IL-10, while OLA can enhance the water solubility and bioavailability of Cur. The experimental results demonstrated that the composite NPs can ameliorate cartilage and subchondral bone damage in mouse OA models, highlighting the therapeutic potential of cytokine-targeted nanomedicines for OA treatment (Figure 14D) [179].

#### Effects of nanomaterials on OA inflammatory microenvironment

The ROS is an important inflammatory mediator produced by the destroyed tissues that induces more protein hydrolase production and leads to ECM degradation. To improve macrophage phenotype and scavenge ROS, Qin *et al.* developed ultrasmall Prussian blue NPs (USPBNPs) to attenuate OA by scavenging ROS and modulating macrophage phenotype. The modified NPs have a diameter of ∼3.5 nm. They can act as both a coating and a reducing agent by adjusting the molecular configuration of the polymer PVP chains. The USPBNPs significantly reduced the secretion of pro-inflammatory cytokines and contributed to converting M1 macrophages into anti-inflammatory M2 macrophages by modulating ROS and oxygen levels in the cellular microenvironment [187]. Besides, Miller *et al.* found that Toll-like receptor (TLR) abnormalities also affect the inflammatory response in OA. Free DNA (cfDNA) in the joint cavity activates immune cells via TLR-9 and promotes the development of OA [188]. Therefore, intervening in OA by targeting cfDNA to NPs or targeting ROS-scavenging antioxidants (e.g. superoxide dismutase, SOD) are potential strategies for treating OA. Recently, Shi *et al.* prepared a PEI-functionalized diselenide-bridged MSN NPs (MSN-PEI) possessing binding of cfDNA and antioxidant properties. Considering the limited joint cavity and dense tissue structure that makes drug delivery challenging, MSN-PEI was sized at 80 nm to balance penetration and retention for optimal therapeutic efficacy. Its positive surface charge promotes the removal of ECM debris such as aggregated proteoglycans or collagen, and reduces the toxicity associated with soluble PEI. These cationic NPs significantly attenuated cartilage degradation and provided potent protection in surgical and collagenase-induced OA models. The mechanism of action of MSN-PEI lies in the inhibition of macrophage M1 polarization by suppressing macrophage M1 polarization, reducing ROS and inhibiting cfDNA-induced inflammation [189]. Recently, Yang *et al.* also developed a drug-delivery system based on copper silicate NPs decorated with polyethylene glycol and loaded with astragaloside-IV (CSP@AS-IV) (Figure 14E). AS-IV is a natural antioxidant, can reduce ROS in OA environments. Cu^2+^ ions in OA settings synergize with AS-IV, jointly exerting antioxidant, antibacterial, anti-inflammatory and chondroprotective effects. In conclusion, these NPs enhance the OA inflammatory environment, decrease ROS, significantly alleviate joint inflammation, downregulate the expression of inflammatory markers and promote cartilage repair [180].

## Summary and outlook

In conclusion, NPs have demonstrated great potential in OA treatment and have made important contributions to improving conventional drugs' toxicity and therapeutic efficacy. This review explores in detail the mechanisms by which the immune system plays a role in OA, and the advantages of nanomaterials in OA treatment and their modulation of the immune response. Nanomaterials have become an ideal tool for OA treatment due to their unique physicochemical properties, degradability and better biocompatibility. Nanomaterials can enhance drug delivery targeting, prolong drug half-life in the OA microenvironment, promote tissue repair and modulate immune responses. Besides, the tunability of nanomaterials allows them to be functionalized according to different therapeutic needs. By tuning the size, shape and surface properties of nanomaterials, precise control of drug release rates can be achieved to improve drug bioavailability. This review systematically delineates the immune cascade mechanisms underlying OA pathogenesis, with particular emphasis on nanotechnology-mediated immune rebalancing through macrophage polarization modulation and proinflammatory cytokine suppression. The engineered surface architectures not only enable spatiotemporal drug release and pathological-site targeting but also integrate dual-functional capabilities for tissue regeneration and immunomodulation via rational ligand conjugation. Nevertheless, critical knowledge gaps persist regarding the dynamic cross-talk within immune networks and the *in vivo* metabolic trajectories of these biomaterials, posing substantial challenges for clinical translation.

However, there are still some challenges to using nanomaterials for OA treatment. Current preclinical investigations on nanomaterial interactions with the immune system predominantly employ human primary cells, primarily synovial fibroblasts or chondrocytes isolated from OA patients undergoing prosthetic replacement surgery. Most experimental protocols utilize passaged cells between passages 3 and 10 post-expansion, with donor populations predominantly aged over 50 years. Notably, critical demographic parameters including sex, smoking history and body mass index remain unstratified in these experimental designs [190]. In contrast, *in vivo* studies predominantly utilize rodent models, with a strong preference for rats and mice as experimental subjects. Notably, these investigations almost exclusively employ male animals, while experimental groups typically comprise 4–9 individuals per cohort. Current therapeutic nanomaterials targeting the immune system for OA management largely overlook variations in treatment efficacy influenced by sex, age, and lifestyle factors. Particular attention should be directed toward sex-specific differences, as emerging evidence highlights their critical role in OA pathogenesis. For instance, studies demonstrate that postmenopausal human chondrocytes cultured with 17β-estradiol and progesterone exhibit reduced senescence markers and enhanced chondrogenic markers. These findings suggest a mechanistic link between menopause-associated cellular aging, ECM degradation and sexually dimorphic OA progression, underscoring the necessity of incorporating sex-based considerations in nanotherapeutic development [191]. Furthermore, preclinical investigations must systematically evaluate how age-related comorbidities (such as osteoporosis and impaired tissue repair capacity) and lifestyle-induced immune dysregulation modulate therapeutic outcomes. Equally critical is addressing technical limitations in current *in vitro* models: standard cell passaging protocols often eliminate pathophysiological stimuli inherent to the OA microenvironment, compromising chondrocyte phenotypic stability. To bridge this gap, advanced model systems capable of recapitulating reversible cartilage degradation under OA-mimetic conditions are urgently needed.

OA management has traditionally focused on symptom relief through pharmacological approaches, primarily targeting pain alleviation. Contemporary research, however, is shifting toward disease-modifying strategies. Emerging nanomaterial-based delivery systems demonstrate therapeutic potential through precise tissue targeting and pathology-responsive drug release, offering advantages in symptom mitigation, cartilage regeneration and functional recovery. Despite promising preclinical outcomes, no nanomaterial formulations have yet received clinical approval for OA treatment. This translational gap underscores the urgent need for accelerating clinical trials of novel nanomaterials while prioritizing cost-effective and biosafe designs. Current challenges necessitate optimization of synthesis protocols to ensure material purity and scalable production. Critical safety concerns persist regarding long-term biostability and potential toxicity profiles, demanding comprehensive *in vivo* evaluations before clinical implementation. Future investigations should adopt a dual focus: elucidating the immunological mechanisms underlying OA pathogenesis while engineering next-generation nanomaterials with enhanced targeting specificity and biocompatibility. The development roadmap requires interdisciplinary efforts to refine material design principles, establish standardized characterization methodologies and validate therapeutic efficacy through robust preclinical models. Such advances could ultimately bridge the gap between nanomaterial innovation and clinical application in OA management.

The clinical translation of nanomaterial-based therapies must address the imperative for personalized therapeutic paradigms. Capitalizing on the inherent tunability of nanomaterials, researchers can engineer functionalized architectures tailored to distinct pathological stages. Future platforms may integrate patient-specific biomarkers and disease profiles to customize treatment regimens, leveraging precise modulation of structural parameters (size, morphology, surface chemistry) to achieve temporal control over drug release kinetics. Such pharmacokinetic optimization could enhance therapeutic precision while minimizing off-target effects. Next-generation intelligent nanomaterials may evolve into multi-functional platforms capable of simultaneous immune cell targeting and inflammatory pathway modulation. For instance, diagnostic–therapeutic combinatorial systems could initially deploy imaging-enabled NPs to map synovial inflammation patterns, followed by precision delivery of immunomodulatory payloads based on real-time pathological feedback. Furthermore, the convergence of nanotechnology and artificial intelligence presents transformative opportunities. AI-integrated diagnostic platforms could process pathobiological signatures captured by nanosensors within affected joints, employing machine learning algorithms to decode disease progression patterns. These AI-powered analytical frameworks may generate predictive models for optimizing treatment protocols, from NP dosage adjustments to dynamic therapeutic regimen adaptations. Such synergetic approaches could catalyze a paradigm shift toward data-driven precision medicine in OA management, where smart nanosystems autonomously respond to evolving disease states through closed-loop biofeedback mechanisms.

Overall, we summarize the impact of the immune system on OA and the hopes and possibilities that nanomaterials offer for OA treatment. We hope that this review will inform future OA treatment techniques.
